# Genome-wide association study for refractive astigmatism reveals genetic co-determination with spherical equivalent refractive error: the CREAM consortium

**DOI:** 10.1007/s00439-014-1500-y

**Published:** 2014-11-04

**Authors:** Qing Li, Robert Wojciechowski, Claire L. Simpson, Pirro G. Hysi, Virginie J. M. Verhoeven, Mohammad Kamran Ikram, René Höhn, Veronique Vitart, Alex W. Hewitt, Konrad Oexle, Kari-Matti Mäkelä, Stuart MacGregor, Mario Pirastu, Qiao Fan, Ching-Yu Cheng, Beaté St Pourcain, George McMahon, John P. Kemp, Kate Northstone, Jugnoo S. Rahi, Phillippa M. Cumberland, Nicholas G. Martin, Paul G. Sanfilippo, Yi Lu, Ya Xing Wang, Caroline Hayward, Ozren Polašek, Harry Campbell, Goran Bencic, Alan F. Wright, Juho Wedenoja, Tanja Zeller, Arne Schillert, Alireza Mirshahi, Karl Lackner, Shea Ping Yip, Maurice K. H. Yap, Janina S. Ried, Christian Gieger, Federico Murgia, James F. Wilson, Brian Fleck, Seyhan Yazar, Johannes R. Vingerling, Albert Hofman, André Uitterlinden, Fernando Rivadeneira, Najaf Amin, Lennart Karssen, Ben A. Oostra, Xin Zhou, Yik-Ying Teo, E. Shyong Tai, Eranga Vithana, Veluchamy Barathi, Yingfeng Zheng, Rosalynn Grace Siantar, Kumari Neelam, Youchan Shin, Janice Lam, Ekaterina Yonova-Doing, Cristina Venturini, S. Mohsen Hosseini, Hoi-Suen Wong, Terho Lehtimäki, Mika Kähönen, Olli Raitakari, Nicholas J. Timpson, David M. Evans, Chiea-Chuen Khor, Tin Aung, Terri L. Young, Paul Mitchell, Barbara Klein, Cornelia M. van Duijn, Thomas Meitinger, Jost B. Jonas, Paul N. Baird, David A. Mackey, Tien Yin Wong, Seang-Mei Saw, Olavi Pärssinen, Dwight Stambolian, Christopher J. Hammond, Caroline C. W. Klaver, Cathy Williams, Andrew D. Paterson, Joan E. Bailey-Wilson, Jeremy A. Guggenheim, The CREAM Consortium

**Affiliations:** 1 National Human Genome Research Institute, National Institutes of Health, 333 Cassell Drive Suite 1200, Baltimore, MD 21224 USA; 2 Department of Epidemiology, Johns Hopkins Bloomberg School of Public Health, Baltimore, MD USA; 3 Wilmer Eye Institute, Johns Hopkins Medical Institutions, Baltimore, MD USA; 4 Department of Twin Research and Genetic Epidemiology, King’s College London, St Thomas’ Hospital Campus, London, UK; 5 Department of Ophthalmology, Erasmus Medical Center, Rotterdam, The Netherlands; 6 Department of Epidemiology, Erasmus Medical Center, Rotterdam, The Netherlands; 7 Singapore Eye Research Institute, Singapore, Singapore; 8 Department of Ophthalmology, Yong Loo Lin School of Medicine, National University of Singapore, Singapore, Singapore; 9 Office of Clinical Sciences, Duke-NUS Graduate Medical School, Singapore, Singapore; 10 Department of Ophthalmology, University Medical Center Mainz, Mainz, Germany; 11 Klinik Pallas, Olten, Switzerland; 12 Medical Research Council Human Genetics Unit, Institute of Genetics and Molecular Medicine, University of Edinburgh, Edinburgh, EH4 2XU UK; 13 Centre for Eye Research Australia, University of Melbourne, Royal Victorian Eye and Ear Hospital, Melbourne, Australia; 14 Centre for Ophthalmology and Visual Science, Lions Eye Institute, University of Western Australia, Perth, Australia; 15 Institute of Human Genetics, Klinikum rechts der Isar, Technische Universität München, Munich, Germany; 16 Department of Clinical Chemistry, Filmlab laboratories, Tampere University Hospital and School of Medicine, University of Tampere, 33520 Tampere, Finland; 17 Statistical Genetics, QIMR Berghofer Medical Research Institute Royal Brisbane Hospital, Brisbane, Australia; 18 Institute of Population Genetics CNR, Traversa La Crucca, 3-07040 Reg. Baldinca, Li Punti, Sassari, Italy; 19 Saw Swee Hock School of Public Health, National University of Singapore, Singapore, Singapore; 20 MRC Integrative Epidemiology Unit (IEU), University of Bristol, Bristol, BS8 2BN UK; 21 School of Social and Community Medicine, University of Bristol, Bristol, BS8 2BN UK; 22 Centre of Epidemiology and Biostatistics, UCL Institute of Child Health, London, UK; 23 Institute of Ophthalmology, University College London, London, UK; 24 Ulverscroft Vision Research Group, UCL Institute of Child Health, London, UK; 25 Genetic Epidemiology, QIMR Berghofer Medical Research Institute Royal Brisbane Hospital, Brisbane, Australia; 26 Beijing Institute of Ophthalmology, Beijing Tongren Hospital, Capital University of Medical Science, Beijing, China; 27 Faculty of Medicine, University of Split, Split, Croatia; 28 Centre for Population Health Sciences, University of Edinburgh, Edinburgh, EH8 9AG UK; 29 Department of Ophthalmology, Sisters of Mercy University Hospital, Zagreb, Croatia; 30 Department of Public Health, Hjelt Institute, University of Helsinki, Helsinki, Finland; 31 Department of Ophthalmology, Helsinki University Central Hospital, Helsinki, Finland; 32 University Heart Center Hamburg, Clinic for general and interventional Cardiology, Hamburg, Germany; 33 Institute for Medical Biometry and Statistics, Universität zu Lübeck, University Hospital Schleswig-Holstein, Campus Lübeck, Lübeck, Germany; 34 DZHK (German Centre for Cardiovascular Research), partner site Hamburg/Kiel/Lübeck, Lübeck, Germany; 35 Dardenne Eye Hospital, Bonn, Germany; 36 Institute of Clinical Chemistry and Laboratory Medicine, University Medical Center Mainz, Mainz, Germany; 37 Department of Health Technology and Informatics, Hong Kong Polytechnic University, Hong Kong SAR, China; 38 Centre for Myopia Research, School of Optometry, Hong Kong Polytechnic University, Hung Hom, Kowloon, Hong Kong SAR, China; 39 Institute of Genetic Epidemiology, Helmholtz Zentrum München, Neuherberg, Germany; 40 Princess Alexandra Eye Pavilion, Edinburgh, EH3 9HA UK; 41 Netherlands Consortium for Healthy Ageing, Netherlands Genomics Initiative, The Hague, The Netherlands; 42 Department of Internal Medicine, Erasmus Medical Center, Rotterdam, The Netherlands; 43 Department of Clinical Genetics, Erasmus Medical Center, Rotterdam, The Netherlands; 44 Department of Statistics and Applied Probability, National University of Singapore, Singapore, Singapore; 45 Department of Medicine, National University of Singapore and National University Health System, Singapore, Singapore; 46 Duke-National University of Singapore Graduate Medical School, Singapore, Singapore; 47 Neuroscience and Behavioural Disorders (NBD) Program, Duke-NUS Graduate Medical School, Singapore, Singapore; 48 Genetics and Genome Biology Program, The Hospital for Sick Children Research Institute, PGCRL Rm 12.9835, 686 Bay Street, Toronto, ON M5G 0A4 Canada; 49 Department of Clinical Physiology, Tampere University Hospital and School of Medicine, University of Tampere, 33521 Tampere, Finland; 50 Research Centre of Applied and Preventive Cardiovascular Medicine, University of Turku, Turku, Finland; 51 Department of Clinical Physiology and Nuclear Medicine, Turku University Hospital, 20041 Turku, Finland; 52 Translational Research Institute, University of Queensland Diamantina Institute, Brisbane, QLD Australia; 53 Division of Human Genetics, Genome Institute of Singapore, Singapore, Singapore; 54 Duke Eye Center, Duke University School of Medicine, Durham, NC USA; 55 University of Sydney, Sydney, Australia; 56 Western Sydney Local Health Network, Sydney, Australia; 57 Westmead Millennium Institute, Westmead, Australia; 58 Ophthalmology and Visual Sciences, Ocular Epidemiology, University of Wisconsin-Madison, 610 North Walnut Street, Room 409, Madison, WI 53726 USA; 59 Beijing Institute of Ophthalmology, Beijing Tongren Eye Center, Beijing Tongren Hospital, Capital Medical University, Beijing Ophthalmology and Visual Science Key Lab, Beijing, China; 60 Department of Ophthalmology, Medical Faculty Mannheim of the Ruprecht-Karls-University of Heidelberg, Mannheim, Germany; 61 Department of Health Sciences and Gerontology Research Center, University of Jyväskylä, Jyväskylä, Finland; 62 Department of Ophthalmology, Central Hospital of Central Finland, Jyväskylä, Finland; 63 University of Pennsylvania School of Medicine, Rm. 314 Stellar Chance Labs, 422 Curie Blvd, Philadelphia, PA 19104 USA; 64 Department of Ophthalmology, King’s College London, St Thomas’ Hospital Campus, London, UK; 65 Dala Lanna School of Public Health, University of Toronto, Toronto, ON Canada

## Abstract

To identify genetic variants associated with refractive astigmatism in the general population, meta-analyses of genome-wide association studies were performed for: White Europeans aged at least 25 years (20 cohorts, *N* = 31,968); Asian subjects aged at least 25 years (7 cohorts, *N* = 9,295); White Europeans aged <25 years (4 cohorts, *N* = 5,640); and all independent individuals from the above three samples combined with a sample of Chinese subjects aged <25 years (*N* = 45,931). Participants were classified as cases with refractive astigmatism if the average cylinder power in their two eyes was at least 1.00 diopter and as controls otherwise. Genome-wide association analysis was carried out for each cohort separately using logistic regression. Meta-analysis was conducted using a fixed effects model. In the older European group the most strongly associated marker was downstream of the neurexin-1 (*NRXN1*) gene (rs1401327, *P* = 3.92E−8). No other region reached genome-wide significance, and association signals were lower for the younger European group and Asian group. In the meta-analysis of all cohorts, no marker reached genome-wide significance: The most strongly associated regions were, *NRXN1* (rs1401327, *P* = 2.93E−07), *TOX* (rs7823467, *P* = 3.47E−07) and *LINC00340* (rs12212674, *P* = 1.49E−06). For 34 markers identified in prior GWAS for spherical equivalent refractive error, the beta coefficients for genotype versus spherical equivalent, and genotype versus refractive astigmatism, were highly correlated (*r* = −0.59, *P* = 2.10E−04). This work revealed no consistent or strong genetic signals for refractive astigmatism; however, the *TOX* gene region previously identified in GWAS for spherical equivalent refractive error was the second most strongly associated region. Analysis of additional markers provided evidence supporting widespread genetic co-susceptibility for spherical and astigmatic refractive errors.

**Electronic supplementary material:**

The online version of this article (doi:10.1007/s00439-014-1500-y) contains supplementary material, which is available to authorized users.

## Introduction

Refractive astigmatism results from the optical summation of the eye’s corneal astigmatism and astigmatism from internal eye components (e.g. lens). In most individuals, these two sources of astigmatism tend to compensate for each other, such that overall refractive astigmatism is typically low in magnitude (Kelly et al. 2004). High levels of refractive astigmatism are usually the result of high corneal astigmatism rather than high internal astigmatism (Keller et al. 1996; Kee 2013). Astigmatism in infancy is a risk factor for amblyopia (Abrahamsson and Sjostrand 2003). In later life, astigmatism commonly accompanies myopia and hyperopia (Mandel et al. 2010; Kee et al. 2005; Farbrother et al. 2004), reducing visual acuity unless corrected by spectacles, contact lenses or refractive surgery (Read et al. 2007).

The results of twin (Dirani et al. 2008; Grjibovski et al. 2006; Parssinen et al. 2012; Teikari and O’Donnell 1989), family (Rakhshani et al. 2012; Mash et al. 1975) and molecular genetic studies (Fan et al. 2011; Lopes et al. 2013; Mackey et al. 2011) suggest that astigmatism is highly heritable, as does its high prevalence in specific ethnic groups such as Native Americans (McKean-Cowdin et al. 2011; Mohindra and Nagaraj 1977; Harvey et al. 2010). For refractive astigmatism, the heritability has been estimated at 0.33 to 0.63 from twin studies (Hammond et al. 2001; Grjibovski et al. 2006; Parssinen et al. 2013). Using a case–control genome-wide association study (GWAS) meta-analysis of 8,513 individuals of Asian ethnicity, Fan et al. (2011) identified the *PDGFRA* gene on chromosome 4q12 as a susceptibility locus for corneal astigmatism. Cases were defined as subjects with corneal astigmatism (averaged between the two eyes) of at least 0.75 diopters (D) and controls as those with corneal astigmatism less than 0.75 D. Three single nucleotide polymorphisms (SNPs) attained genome-wide significance (*P* < 5.0E−08); rs7677751, rs2307049 and rs7660560. SNPs in the same region of *PDGFRA* have since been found to be associated with both corneal curvature and axial length (Han et al. 2011; Guggenheim et al. 2013; Mishra et al. 2012), but not with spherical refractive error (Guggenheim et al. 2013). A second GWAS meta-analysis in 22,100 individuals of European descent by Lopes et al. (2013) reported suggestive evidence that SNPs in the *VAX2* gene on chromosome 2p13 also confer susceptibility to refractive astigmatism (most strongly associated SNP, rs3771395; *P* = 2.0E−07). These authors modelled astigmatism as a continuous trait, using an inverse normal transformation of the refractive astigmatism averaged between the two eyes.

The GWAS meta-analyses of Fan et al. (2011) and Lopes et al. (2013) both assessed large numbers of individuals derived from cohorts that were largely population based. It is therefore unlikely that common autosomal genetic variants, i.e. those with a minor allele frequency (MAF) >5 %, with profound effects on the risk of developing astigmatism (e.g. OR > 2) exist, as both studies would have had high power to detect them. Instead, the results of the two studies imply that most of the additive genetic risk for astigmatism arises from the combined action of a large number of individual risk variants, each with a small effect. This scenario, which also holds for spherical refractive error (Solouki et al. 2010; Hysi et al. 2010; Verhoeven et al. 2013b; Kiefer et al. 2013), suggests that substantially increasing the sample size of GWAS meta-analyses will be an effective method of discovering new variants, albeit with increasingly diminishing returns (Lango Allen et al. 2010). Here, we describe the largest GWAS for refractive astigmatism yet undertaken involving almost 46,000 persons.

## Methods

### Selection of studies for inclusion in the meta-analysis

The CREAM consortium comprises researchers from more than 30 research groups who share a common interest in the genetics of refractive error. From March to July 2012, all Principal Investigators (PIs) of studies known to CREAM members who had collected refractive error phenotype information and genome-wide genotyping information on a study sample were invited to join CREAM. An analysis plan detailing the protocol for the astigmatism GWAS meta-analysis was circulated, inviting all PIs to perform the requested analyses and to submit GWAS results for their study sample. There were no restrictions on which studies were eligible to join the meta-analysis.

### Study cohorts and meta-analysis overview

GWAS results were meta-analysed for a total of 32 cohorts. The subject demographics of the cohorts are summarised in Table 1: Further details can be found in the Supplement and the previous publications (Rahi et al. 2011; Fraser et al. 2012; Boyd et al. 2013; Vitart et al. 2010; Parssinen et al. 2010; Sperduto et al. 1996; Foong et al. 2007; Foran et al. 2003; Cornes et al. 2012; Hofman et al. 2011; Burdon et al. 2011; Khor et al. 2011; Oexle et al. 2011; Vithana et al. 2011; Paterson et al. 2010; Klein et al. 2010; Mackey et al. 2009; Nelis et al. 2009; Raitakari et al. 2008; Spector and Williams 2006; Wichmann et al. 2005; Pardo et al. 2005; Aulchenko et al. 2004; Clemons et al. 2003; Kassoff et al. 1999, 2001; Mitchell et al. 1995; Shamoon et al. 1993). The mean age of the participants in each cohort varied from 15 to 74 years and 37,608 of them were of White European ancestry while 10,212 were of Asian ancestry. Because the magnitude and axis of astigmatism are known to vary with age (Anstice 1971; Lyle 1971), and to limit the effects of differing SNP-causal variant relationships across ethnicities, meta-analyses were carried out separately for (a) White Europeans aged <25 years, (b) White Europeans aged ≥25 years, and (c) Asians aged ≥25 years. This age classification scheme follows that adopted previously by the CREAM consortium (Verhoeven et al. 2013a, b), and was agreed to by the CREAM Executive Committee prior to commencement of the meta-analyses. A final meta-analysis was performed combining all independent samples from these three groups with the SCORM study of Asians aged <25 years. Each participating study defined the astigmatism trait in the same manner and performed association analyses specifically for this study using equivalent logistic regression models (described below and in the supplement).

**Table 1:** Cohort demographics

Study	Ethnicity	*N* (cases/controls)	Age, years (mean ± SD)	Astigmatism, D (mean ± SD)	Astigmatism median, D (IQR)	Astigmatism, D (range)	% Female
European adult cohorts							
1958 British Birth Cohort	White European	1,645 (182/1,463)	42 ± 0	0.47 ± 0.53	0.38 (0.13–0.63)	0.00–4.50	45.8
ALSPAC mothers	White European	1,889 (343/1,546)	44 ± 2	0.63 ± 0.53	0.50 (0.25–0.75)	0.00–4.62	100.0
AREDS	White European	1,864 (567/1,297)	68 ± 5	0.77 ± 0.67	0.75 (0.25–1.00)	0.00–4.50	59.2
BATSplusTEST	White European	204 (49/155)	40 ± 14	0.63 ± 0.57	0.38 (0.25–0.89)	0.00–2.75	62.7
CROATIA-Korcula	White European	826 (135/691)	56 ± 13	0.63 ± 0.52	0.50 (0.25–0.75)	0.00–4.00	64.7
CROATIA-Split	White European	343 (35/308)	51 ± 13	0.55 ± 0.41	0.44 (0.25–0.63)	0.00–3.00	56.3
CROATIA-Vis	White European	529 (104/425)	56 ± 13	0.68 ± 0.57	0.51 (0.21–0.81)	0.00–4.68	59.7
ERF4	White European	2,485 (472/2,013)	49 ± 14	0.58 ± 0.54	0.50 (0.25–0.75)	0.00–4.13	43.4
FITSA	White European	87 (18/69)	68 ± 3	0.75 ± 0.52	0.63 (0.38–0.88)	0.00–3.50	100.0
Framingham	White European	1,532 (745/787)	60 ± 12	0.78 ± 0.56	0.63 (0.38–1.00)	0.00–4.38	56.1
GUTENBERG	White European	3,954 (640/3,314)	56 ± 11	0.55 ± 0.54	0.44 (0.13–0.75)	0.00–4.63	49.2
KORA	White European	1,852 (448/1,404)	56 ± 12	0.72 ± 0.64	0.50 (0.25–1.00)	0.00–4.75	50.6
OGLIASTRA	White European	472 (49/423)	52 ± 16	0.31 ± 0.52	0.00 (0.00–0.50)	0.00–3.00	69.0
ORCADES	White European	502 (113/389)	58 ± 14	0.70 ± 0.65	0.56 (0.22–0.90)	0.00–4.69	56.8
ROTTERDAM 1	White European	5,422 (2,193/3,229)	69 ± 9	0.95 ± 0.66	0.75 (0.38–1.13)	0.00–4.75	58.6
ROTTERDAM 2	White European	1,973 (725/1,248)	64 ± 7	0.89 ± 0.59	0.75 (0.44–1.07)	0.00–4.50	54.3
ROTTERDAM 3	White European	1,971 (580/1,391)	56 ± 6	0.81 ± 0.57	0.63 (0.31–0.94)	0.00–4.00	56.5
TwinsUK	White European	2,658 (751/1,907)	55 ± 13	0.80 ± 0.65	0.63 (0.38–1.00)	0.00–4.88	91.1
WESDR adults	White European	280 (69/211)	35 ± 8	0.71 ± 0.65	0.50 (0.19–0.81)	0.00–4.50	75.4
YFS	White European	1,480 (269/1,211)	42 ± 5	0.64 ± 0.52	0.50 (0.25–0.75)	0.00–4.13	55.3
Asian adult cohorts							
BES	Chinese	585 (154/431)	62 ± 9	0.66 ± 0.59	0.50 (0.25–1.00)	0.00–3.50	65.8
HK-MGS adults	Chinese	120 (59/61)	34 ± 7	1.29 ± 1.05	0.97 (0.50–1.84)	0.00–5.31	61.7
SCES	Chinese	1,662 (670/992)	57 ± 9	0.99 ± 0.63	0.85 (0.48–1.23)	0.00–4.30	48.8
SIMES	Malay	2,165 (706/1,459)	57 ± 11	0.90 ± 0.66	0.73 (0.39–1.06)	0.00–4.85	50.8
SINDI	Indian	1,998 (739/1,259)	56 ± 9	0.96 ± 0.62	0.83 (0.47–1.18)	0.00–4.53	48.7
SP2	Chinese	1,954 (543/1,411)	48 ± 11	0.81 ± 0.56	0.68 (0.36–0.99)	0.00–4.18	54.2
STARS	Chinese	811 (205/606)	39 ± 5	0.72 ± 0.67	0.60 (0.21–0.94)	0.00–4.32	48.0
European youngsters cohorts							
ALSPAC children	White European	3,828 (580/3,248)	15 ± 0.3	0.65 ± 0.42	0.63 (0.38–0.75)	0.00–4.25	48.8
BATSplusTEST children	White European	561 (60/501)	18 ± 4	0.40 ± 0.48	0.25 (0.13–0.5)	0.00–4.00	54.0
RAINE	White European	1,007 (215/792)	20 ± 0	0.74 ± 0.40	0.69 (0.45-0.93)	0.08–3.11	49.3
WESDR children	White European	244 (52/192)	18 ± 4	0.64 ± 0.57	0.50 (0.25–0.75)	0.00–3.38)	50.8
Asian youngsters cohort							
SCORM	Chinese	917 (247/670)	11 ± 1	0.77 ± 0.66	0.57 (0.21–0.94)	0.00–4.32	48.0

### Phenotypic assessment

Subjects underwent an ophthalmic examination that included either subjective refraction, cycloplegic autorefraction or non-cycloplegic autorefraction (Supplemental Methods and Supplemental Table S1a). Astigmatism was defined in the same way during association analysis in all cohorts participating in this meta-analysis study. Participants with conditions that could alter refraction, such as cataract surgery, laser refractive procedures, retinal detachment surgery, keratoconus or ocular or systemic syndromes were excluded. Additional exclusion criteria were, firstly, a cylinder power ≥5.00 D in either eye (to exclude subjects with undiagnosed keratoconus or potential measurement errors), and secondly, a difference in cylinder power between the two eyes beyond four standard deviations from the mean (except for subjects with data for only one eye). Subjects were classified as astigmatic cases if the average cylinder power in the two eyes was ≥1.00 D and as controls otherwise (note that cylinder axis was ignored). The threshold value of 1.00 D was chosen due to its common usage in prior work (Read et al. 2007; Huynh et al. 2007). The average of the two eyes was taken to maximise statistical power (Carbonaro et al. 2009).

### Genotyping and genotype imputation

Genotyping and imputation were carried out as described previously (Verhoeven et al. 2013b). In brief, participants in each cohort were genotyped using a whole genome SNP platform. The genotypes of subjects that passed a series of quality control (QC) filters, including call rate at least >95 % and ancestry matching that of the reference population, were imputed to a common set of markers (HapMap Phase 2) with either MACH (Li et al. 2010) or IMPUTE (Howie et al. 2012). SNPs that passed cohort-specific QC metrics were used as a framework for imputation, and reference haplotypes were chosen from the best available HapMap Phase 2 ancestry group (Verhoeven et al. 2013b). See Supplemental Methods and Table S1b for more details.

### Statistical analysis

A GWAS was carried out separately for each participant cohort. SNPs were tested individually for association with astigmatism in a logistic regression model, with case/control status as the dependent variable. SNP imputed dosage was modelled as a linear covariate (on a continuous scale from 0 to 2) where one allele was assigned as the reference allele and the other allele the risk allele. Age and sex were included as additional covariates when appropriate. If significant population stratification was detected in a cohort, then either the first two principal components (PCs) were included in the logistic regression or an analysis method was used that jointly adjusted for population stratification and cryptic relatedness as part of the analysis. This approach is commonly used in GWAS meta-analysis (Eeles et al. 2009; Chen et al. 2012; Wang et al. 2012). Details of the GWAS analyses performed in each cohort are given in Supplemental Methods. SNPs were carried forward for meta-analysis if they met the following criteria of a MAF >1 %, and an OR (odds ratio) between 0.2 and 5.0 (the latter step being included to remove SNPs with an OR of approximately zero or infinity, which occurred for a few SNPs in the smaller cohorts due to low minor allele counts). Effect estimates were reported with reference to the positive strand of the NCBI Build 36 reference sequence of the human genome. Meta-analysis was carried out using a fixed effects model with METAL (Willer et al. 2010). For the meta-analysis of all cohorts, the adult ALSPAC sample was excluded because, given the inclusion of the ALSPAC young persons sample (biological relatives of the adults), this could have led to falsely inflated estimates of association. The number of subjects contributing information to the meta-analysis summary statistic varied, as shown in Tables 2 and 3. This occurred primarily through markers being monomorphic (uninformative) in certain samples, and to a small extent through missing data for certain markers in specific individuals. A *P* value <5.0E−08 was used to declare genome-wide significance (Dudbridge and Gusnanto 2008; Evangelou and Ioannidis 2013).

**Table 2:** Most strongly associated SNPs in the 3 meta-analyses

SNP	Chr	Pos	RA	NRA	RAF (min–max)	OR	95 % CI	*P* value	*I* ^2^	*N*	Gene(s)
European subjects aged ≥25 years											
rs1401327	2	49900987	A	G	0.113–0.174	1.157	1.098–1.218	3.92E−08	0	31,694	*NRXN1*
rs17795388	2	49900356	G	A	0.113–0.174	1.157	1.098–1.218	4.16E−08	0	31,691	*NRXN1*
rs11690625	2	49908115	C	A	0.113–0.175	1.156	1.098–1.218	4.17E−08	0	31,731	*NRXN1*
rs17795358	2	49897928	A	G	0.113–0.173	1.156	1.097–1.218	4.94E−08	0	31,672	*NRXN1*
rs925931	2	49913312	C	T	0.113–0.173	1.148	1.090–1.210	2.06E−07	0	31,727	*NRXN1*
rs885560	2	49909442	G	A	0.113–0.175	1.146	1.088–1.207	2.46E−07	0	31,728	*NRXN1*
rs6708111	2	49878453	A	C	0.102–0.168	1.139	1.082–1.200	7.27E−07	0	31,531	*NRXN1*
rs11690252	2	49890187	T	G	0.230–0.342	1.105	1.060–1.151	2.59E−06	0	31,511	*NRXN1*
rs1878856	2	49877706	C	T	0.214–0.336	1.105	1.059–1.153	3.56E−06	0	31,603	*NRXN1*
rs12638075	3	1.42E + 08	C	T	0.014–0.024	1.376	1.200–1.577	4.69E−06	0	27,304	*TRIM42/CLSTN2*
rs2309717	4	27859336	A	C	0.089–0.170	1.143	1.083–1.206	1.02E−06	11.8	31,143	*STIM2/PCDH7*
rs2871434	4	29931147	T	A	0.095–0.154	1.140	1.079–1.204	2.66E−06	13.6	31,664	*STIM2/PCDH7*
rs12212674	6	22195053	A	T	0.496–0.621	1.099	1.058–1.142	1.45E−06	0	31,691	*LINC00340*
rs6901423	6	22194271	G	A	0.496–0.621	1.099	1.057–1.142	1.63E−06	0	31,689	*LINC00340*
rs4712652	6	22186594	A	G	0.495–0.687	1.097	1.055–1.141	3.13E−06	0	28,910	*LINC00340*
rs9366427	6	22204592	G	C	0.487–0.619	1.094	1.053–1.136	4.15E−06	0	31,773	*LINC00340*
rs4799964	18	26239477	G	T	0.020–0.048	1.267	1.152–1.394	1.16E−06	0	31,881	*MIR302F*
rs12607243	18	26229228	G	A	0.020–0.050	1.264	1.149–1.392	1.60E−06	0	31,882	*MIR302F*
European subjects aged <25 years											
rs6688613	1	165218493	T	C	0.240–0.253	1.309	1.170–1.465	2.68E−06	0	5,640	*MAEL*
rs1327866	1	165219534	G	A	0.238–0.253	1.308	1.169–1.464	2.89E−06	0	5,640	*MAEL*
rs7550698	1	165217705	C	T	0.240–0.253	1.308	1.168–1.463	3.02E−06	0	5,640	*MAEL*
rs7528849	1	165221494	G	A	0.240–0.253	1.307	1.168–1.463	3.11E−06	0	5,640	*MAEL*
rs7518155	1	165221520	G	T	0.240–0.253	1.307	1.168–1.462	3.19E−06	0	5,640	*MAEL*
rs7545911	1	165214305	A	G	0.240–0.253	1.309	1.169–1.467	3.35E−06	0	5,640	*MAEL*
rs6682062	1	165216603	C	G	0.240–0.253	1.309	1.168–1.467	3.39E−06	0	5,640	*MAEL*
rs2296837	1	165225225	C	T	0.240–0.253	1.305	1.166–1.461	3.53E−06	0	5,640	*MAEL*
rs11578336	1	165225334	G	T	0.240–0.253	1.304	1.166–1.460	3.71E−06	0	5,640	*MAEL*
rs1366200	5	115349718	G	T	0.312–0.321	1.308	1.174–1.457	1.04E−06	48.7	5,640	*AQPEP*
rs17712049	7	48236741	C	T	0.875–0.904	1.569	1.295–1.902	4.39E−06	0	5,640	*ABCA13*
rs13257518	8	32755116	A	T	0.177–0.217	1.370	1.202–1.561	2.36E−06	15.7	5,640	*NRG1*
rs10503929	8	32733525	C	T	0.167–0.215	1.352	1.192–1.534	2.68E−06	32.3	5,640	*NRG1*
rs2975500	8	32724907	A	G	0.110–0.161	1.435	1.231–1.673	3.95E−06	0	5,640	*NRG1*
Asian adults											
rs7534824	1	101394034	A	G	0.967–0.974	2.304	1.651–3.214	9.00E−07	0	4,812	*LOC101928334*
rs10496034	2	54998439	C	G	0.170–0.287	1.216	1.122–1.318	2.13E−06	0	8,780	*EML6*
rs428445	20	54469954	T	G	0.713–0.954	1.314	1.175–1.470	1.84E−06	0	8,908	*CASS4/GCNT7*
rs6999	20	54527308	A	G	0.713–0.957	1.303	1.164–1.459	4.30E−06	0	8,904	*CASS4/GCNT7*

The table shows all SNPs with *P* < 5.0E−06

*RA* risk allele, *NRA* non–risk (reference) allele, *RAF* risk allele frequency in each cohort, *OR* odds ratio, *95* *%* *CI* 95 % confidence interval of odds ratio, *I* ^*2*^ heterogeneity statistic, *N* total sample size

**Table 3:** Most strongly associated SNPs in the meta–analysis of all cohorts

SNP	Chr	Pos	RA	NRA	RAF (min–max)	OR	95 % CI	*P* value	*I* ^2^	*N*	Gene
rs1401327	2	49900987	A	G	0.113–0.174	1.139	1.084–1.198	2.93E−07	0	35,445	NRXN1
rs11690625	2	49908115	C	A	0.113–0.175	1.139	1.084–1.197	2.95E−07	0	35,482	NRXN1
rs17795388	2	49900356	G	A	0.113-0.174	1.139	1.084–1.198	3.10E−07	0	35,442	NRXN1
rs17795358	2	49897928	A	G	0.113–0.173	1.139	1.083–1.197	3.67E−07	0	35,423	NRXN1
rs925931	2	49913312	C	T	0.010–0.173	1.125	1.071–1.182	2.64E−06	3.3	39,567	NRXN1
rs885560	2	49909442	G	A	0.010–0.175	1.123	1.069–1.179	3.46E−06	5.5	39,566	NRXN1
rs6708111	2	49878453	A	C	0.102–0.168	1.124	1.069–1.182	4.42E−06	0	35,282	NRXN1
rs7581641	2	8543557	T	C	0.012–0.103	1.225	1.123–1.336	4.74E−06	0	41,865	NRXN1
rs6892230	5	65175520	A	G	0.016–0.078	1.236	1.133–1.349	1.87E−06	41.3	37,591	NLN
rs12212674	6	22195053	A	T	0.134–0.621	1.086	1.050–1.123	1.49E−06	0	45,134	LINC00340
rs6901423	6	22194271	G	A	0.134–0.621	1.083	1.048–1.120	3.00E−06	0	45,132	LINC00340
rs1034071	6	22205354	C	T	0.137–0.608	1.081	1.046–1.118	3.73E−06	0	45,330	LINC00340
rs7823467	8	60241288	T	C	0.388–0.713	1.085	1.052–1.120	3.47E−07	22.9	45,273	TOX
rs10086929	8	60252851	A	G	0.430–0.709	1.083	1.049–1.118	7.36E−07	22.3	45,156	TOX
rs6471768	8	60230697	T	A	0.435–0.710	1.082	1.048–1.117	1.07E−06	23.9	45,125	TOX
rs4531042	8	60251242	G	A	0.388–0.737	1.082	1.048–1.118	1.45E−06	32.9	45,277	TOX
rs4738757	8	60218783	A	G	0.388–0.701	1.080	1.046–1.115	1.89E−06	26.9	45,122	TOX
rs12675886	8	60309643	C	T	0.458–0.704	1.079	1.045–1.114	2.50E−06	14.3	45,082	TOX
rs6997378	8	60330443	T	G	0.460–0.705	1.077	1.043–1.111	4.95E−06	17.3	45,085	TOX
rs1944146	11	130195372	A	G	0.524–0.608	1.080	1.046–1.115	2.62E−06	17.3	45,243	LOC100507431
rs7934985	11	130194532	G	A	0.523–0.613	1.080	1.046–1.116	2.66E−06	4.8	45,123	LOC100507431

The table shows all SNPs with *P* < 5.0E−06

## Results

Meta-analyses of refractive astigmatism GWAS results were carried out for three subject groups: White Europeans aged ≥25 years, White European subjects aged <25 years, and Asians aged ≥25 years. There was little evidence of population stratification in any of the meta-analysis results (Genomic Control lambda, *λ*_GC_ = 1.014, 1.011, 1.018 and 1.022 for White Europeans aged ≥25 years, White European subjects aged <25 years, Asians aged ≥25 years, and all samples combined, respectively).

### Meta-analysis of White Europeans aged at least 25 years

**Fig. 1:**
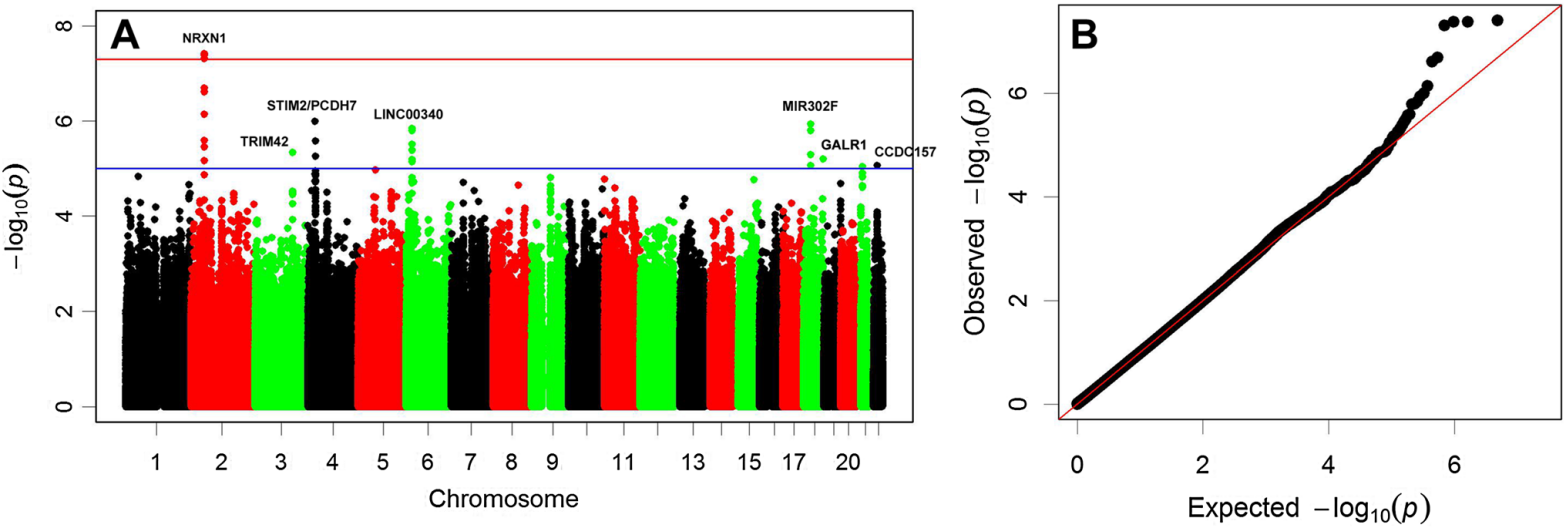
Results of the meta-analysis of White European subjects aged ≥25 years old. **a** Manhattan plot of log *P* values against genomic position. The *red horizontal line* is the threshold commonly used to for declaring genome-wide significance (*P* = 5.0E−08). The *blue line indicates* *P* = 1.0E−05. Genes adjacent to the association signal are indicated. **b** Quantile–quantile (QQ) plot of observed versus expected distribution of log *P* values. The *red line* shows the distribution expected by chance

**Fig. 2:**
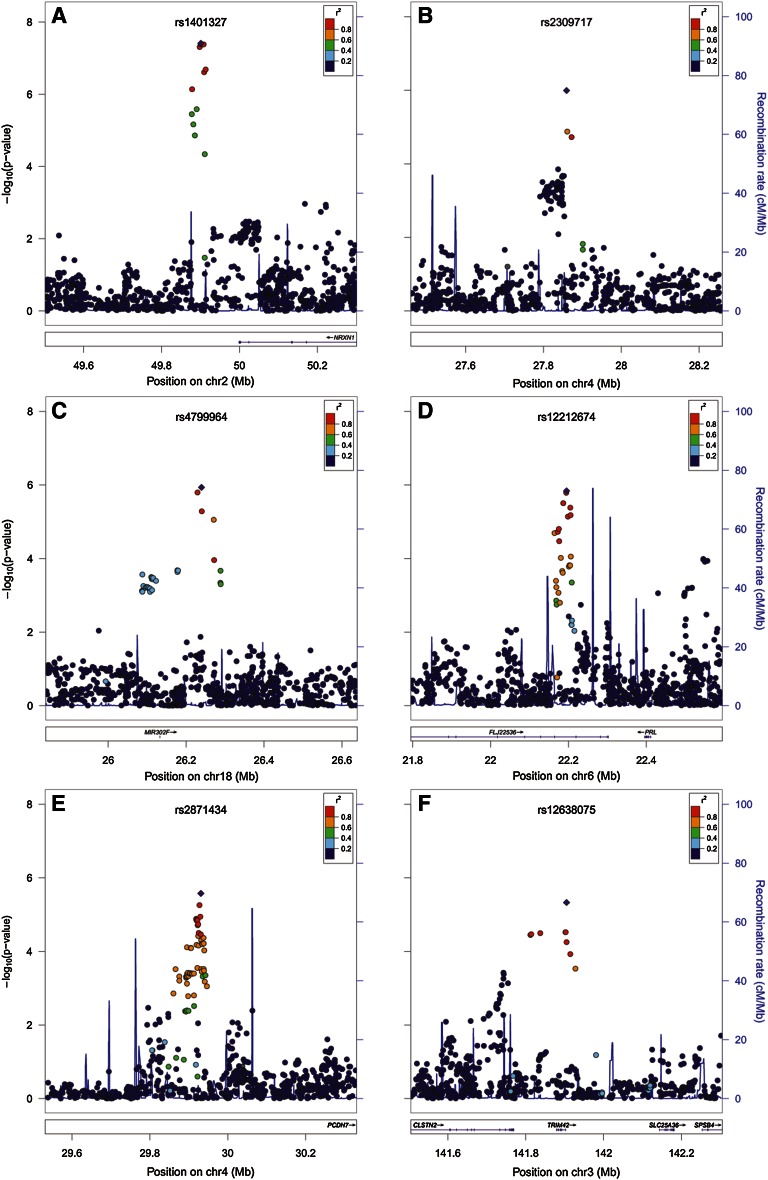
Regions showing the strongest evidence for association with refractive astigmatism in the meta-analysis of White Europeans aged ≥25 years

For the meta-analysis of older White European individuals (*N* = 31,968) there were six regions containing markers with *P* values <5.0E−06, suggestive of association (Table 2; Figs. 1, 2). However, only a single region contained markers that met the *P* value conventionally accepted to declare genome-wide significance (*P* < 5.0E−08). This was at 2p16.3, downstream of the gene encoding neurexin-1 (*NRXN1*; Fig. 2a) with the most strongly associated marker being rs1401327. Each copy of the A allele of rs1401327 increased the odds of astigmatism with an OR 1.16 (95 % CI 1.10 to 1.22; *P* = 3.92E−08). The next most strongly associated regions were at 3q23, 4p15, 6p22.3, and 18q12.1 (Table 2). There was little evidence of heterogeneity of effect across cohorts at any of the above loci (*I* ^2^ < 14; Table 2).

### Meta-analysis of White Europeans aged less than 25 years

The meta-analysis of younger White European cohorts identified four regions with *P* values below 5.0E−06 (Table 2). However, the much smaller sample size (*N* = 5,640) meant that this meta-analysis had limited statistical power to detect true-positive associations. The most strongly associated SNP was rs1366200 (OR 1.31, 95 % CI 1.17–1.46; *P* = 1.04E−06) within the *AQPEP* gene on chromosome 5q23.1.

### Meta-analysis of Asians aged at least 25 years

In the meta-analysis of Asian cohorts (*N* = 9,295) the most strongly associated marker was rs7534824 (OR 2.30, 95 % CI 1.65 to 3.22; *P* 9.00E−07) within a gene of unknown function, *LOC101928334*, on chromosome 1. This marker had a low allele frequency (MAF = 0.03). Two other regions also contained SNPs with *P* values <5.0E−06 (Table 2). However, this meta-analysis also had limited statistical power to detect true-positive associations.

### Meta-analysis of all cohorts

To search for evidence to corroborate the initial findings, we carried out a meta-analysis of all independent individuals from the above three cohort groups combined with Asians <25 years of age from the SCORM study (*N* = 45,931). As shown in Table 3, this revealed little evidence across cohort groups to substantiate the initial findings. The three most strongly associated regions were the *NRXN1* locus, the *TOX* gene locus on chromosome 8q12.1, and the *LINC00340* gene locus at 6p22.3, all of which were amongst the most highly associated regions identified in the meta-analysis of older White European subjects. Association at the *NRXN1* gene locus (rs1401327, OR 1.139, 95 % CI 1.084–1.198, *P* = 2.93E−07) was driven solely by the European cohorts, since the associated SNPs were monomorphic in Asians, and thus uninformative. The most strongly associated marker at the *TOX* gene locus was rs7823467 (OR 1.09, 95 % CI 1.05–1.12; *P* = 3.47E−07) while that at the *LINC00340* gene locus was rs12212674 (OR 1.09, 95 % CI 1.05–1.12; *P* = 1.49E−06).

**Fig. 3:**
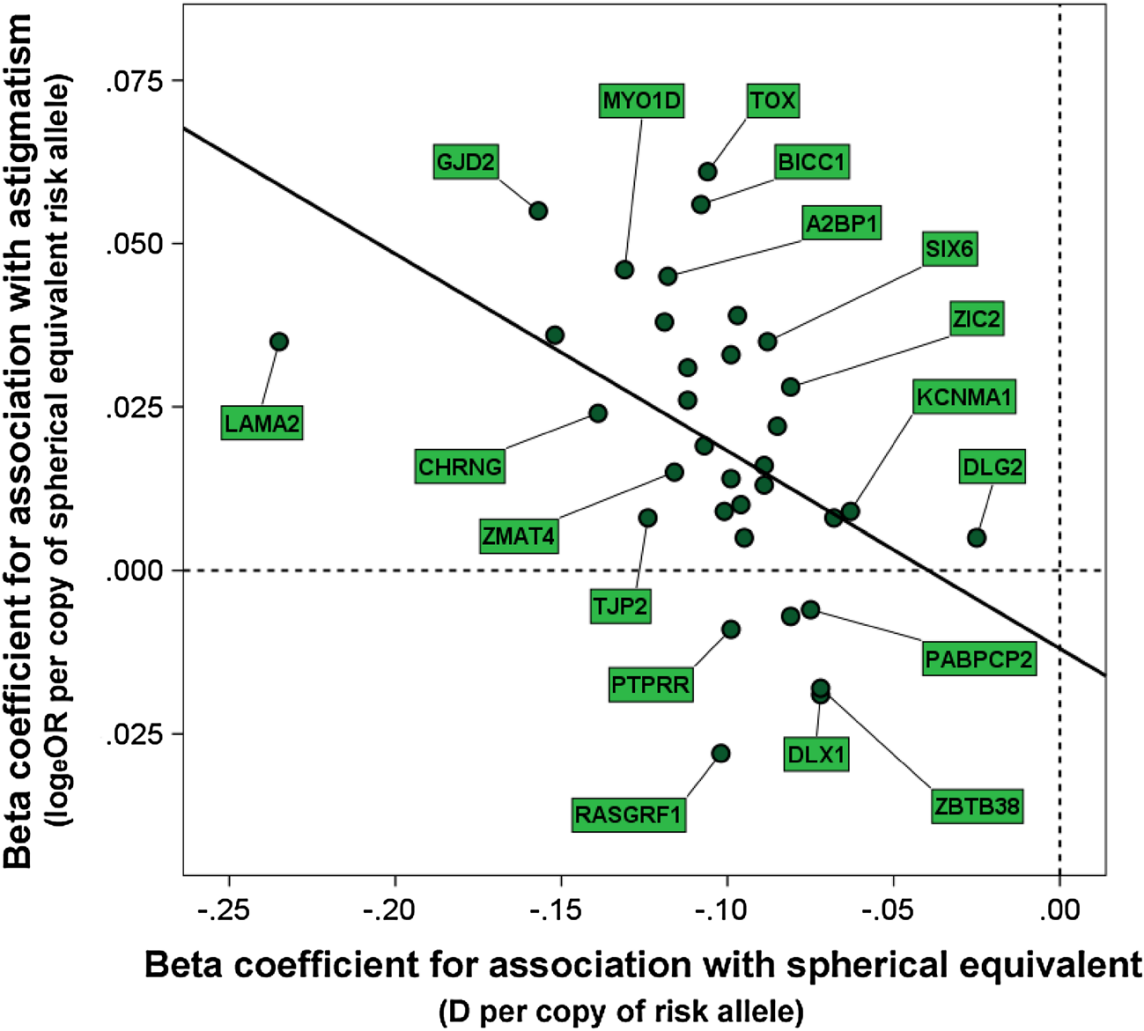
Common genetic determinants for spherical equivalent refractive error are shared with refractive astigmatism. GWAS meta-analysis beta coefficients (which quantify the effect size of SNPs) were compared between studies of spherical equivalent and refractive astigmatism. The SNP beta coefficients for spherical equivalent were obtained from the CREAM consortium GWAS for spherical equivalent (Verhoeven et al. 2013b), while those for refractive astigmatism were from the current study. The 34 SNPs analysed were chosen based on prior genome-wide significant evidence of association with spherical equivalent in the CREAM (Verhoeven et al. 2013b) and 23andMe (Kiefer et al. 2013) GWAS meta-analyses. The *solid line* is the best linear fit to the data

Interestingly, the *TOX* region is one of the loci identified in the CREAM consortium GWAS for spherical equivalent refractive error (Verhoeven et al. 2013b) and the age of onset of myopia GWAS carried out by 23andMe (Kiefer et al. 2013). Therefore, to investigate whether spherical refraction and astigmatism share common genetic determinants more widely, we examined the association with refractive astigmatism of 34 genome-wide significant SNPs (Table S1) reported in the CREAM (Verhoeven et al. 2013b) and 23andMe (Kiefer et al. 2013) spherical equivalent GWAS meta-analyses (4 additional SNPs associated with spherical equivalent could not be included since they were not analysed in the current study). For each SNP, the effect size (beta coefficient describing the magnitude of association) with spherical equivalent was plotted against the effect size for association with refractive astigmatism (Fig. 3). The betas were found to be highly correlated (*r* = −0.59, *P* = 2.10E−04). Excluding the SNP in the *TOX* gene region had minimal influence on the correlation of the betas for the remaining 33 SNPs (*r* = −0.60, *P* = 2.29E−04).

## Discussion

This GWAS meta-analysis of nearly 46,000 individuals identified several novel, suggestive candidate genes/regions for refractive astigmatism, including *NRXN1*, *TOX* and *LINC00340*. One of these regions, near the *NRXN1* gene region, reached genome-wide significance in the White European adult group. Two-thirds of the ~46,000 subjects included in the full meta-analysis were White European adults and so the results are likely to have been driven mainly by this group. Therefore, until the opportunity arises for replication in independent samples, especially in large numbers of comparable White European adults, caution is needed in interpreting these results. These results should not be considered to be relevant to other populations until replicated in younger White European samples or in other ethnic groups.

### Novel candidate genes underlying the observed associations

Neurexin-1, one of the largest genes in the human genome, is thought to function in cell adhesion, as well as synapse development and maintenance (Kirov et al. 2008, 2009). Structural genomic deletions that delete or disrupt *NXRN1* are strongly implicated in causing psychiatric and cognitive phenotypes including schizophrenia, autism and mental retardation (Bena et al. 2013). To our knowledge, these conditions are not known to be associated with refractive astigmatism (although refractive errors, in general, are more prevalent in individuals with learning difficulties, Woodhouse et al. 2003). A recent survey of 25 patients with exonic deletions involving the gene for neurexin-1 (Bena et al. 2013) unfortunately did not describe these patients’ ocular features. While the strength of association reached genome-wide significance in the adult European sample (*N* = 31,968, *P* = 3.92E−08), this weakened when the younger European subjects were included (*N* = 35,719, *P* = 2.93E−07) while having little impact on the estimated effect size (OR 1.16 and 1.14, respectively). The associated SNPs in this region were monomorphic in Asian subjects, suggesting they arose relatively recently in human evolution.

The associated variants at 8q12.1 lie upstream of the *TOX* promoter and thus would be well placed to influence its transcription level. However, it is not clear whether *TOX* or a nearby gene mediates this locus’ impact on spherical equivalent refractive error, and potentially astigmatism. The known roles of *TOX* relate to immune function, which argues against a role in refractive development and instead suggests that another gene such as *SDCBP* (syndecan-binding protein) also known as syntenin, which lies 600 kb from the most strongly associated marker may be involved. Syntenin acts as a link between the proteoglycan/matrix receptor syndecan-1 and the cytoskeleton, and its proposed functions include cell adhesion. Furthermore, syntenin-null mice show wound healing defects that are particularly marked in the cornea (Stepp et al. 2002, 2010).

The 6p22.3 locus containing the long intergenic non-coding RNA gene *LINC00340* (also known as *FLJ22536* and *CASC15*) is gene poor (Fig. 2d) yet has previously shown association with aggressive neuroblastoma in GWAS studies (Capasso et al. 2013). The mechanisms through which non-coding RNAs act are poorly understood (Guttman et al. 2009; Gibb et al. 2011) but in the case of lincRNAs the mechanism may involve epigenetic regulation (Salta and De Strooper 2012). No obvious candidate astigmatism susceptibility gene is present in this genomic location. As with *NRXN1*, the association with *LINC00340* was almost wholly driven by the adult European cohorts (*P* = 1.45E−06 versus *P* = 1.49E−06 in all cohorts combined).

As well as *NRXN1* and *SDCBP*, additional genes in the most strongly associated regions have putative roles in cell adhesion and/or synapse function. The gene nearest to the lead SNP at 3q23 in European adults (rs12638075, *P* = 4.69E−06) is *TRIM42* (tripartite motif containing-42). Because members of the *TRIM* gene family function mostly in immune signalling (Versteeg Gijs et al. 2013), the adjacent gene *CLSTN2* (calsyntenin-2; also known as cadherin-related family member-13) is potentially of greater interest given its proposed role in cell adhesion and synapse function (Preuschhof et al. 2010). Furthermore, the association described above for markers in the vicinity of the *SDCBP* gene, encoding syntenin, lends support to the putative involvement of *CLSTN2*. One of the two regions on chromosome 4p15 (lead SNP rs2871434; Fig. 2e) contains the *PCDH7* (protocadherin-7) gene, which given its role in cell adhesion is a plausible candidate gene for astigmatism. In mice homozygous for a null allele of the *EGR1* gene, which develop a transient axial myopia postnatally, a member of the protocadherin gene family, *Pcdhb9*, was the most highly differentially expressed retinal gene when compared to wild-type mice (Schippert et al. 2009). The second associated region at 4p15 (lead SNP rs2309717; Fig. 2b) contains no known genes, the closest being *MIR4275*, which lies 600 kb away. However, amongst the more than 6,000 predicted targets of miR-4275 is the nearby *PCDH7*.

### Genetic co-determination of spherical equivalent and refractive astigmatism

One of the most exciting findings from this study was the evidence for overlap in genetic susceptibility between spherical and astigmatic refractive errors (Fig. 3). It is well known that spherical and astigmatic refractive errors tend to co-occur (Read et al. 2007; Guggenheim and Farbrother 2004). However, to our knowledge this is the first study to provide evidence supporting shared genetic susceptibility for the two traits. Kee and Deng (2008) and Kee et al. (2005) have shown in monkeys and chickens that visual experience can alter spherical equivalent and astigmatic refractive errors concurrently. Hence, in line with the view that genetic factors might alter refractive development by regulating how the eye responds to visual cues (Chen et al. 2011; Wallman 1994), it is feasible that causal variants tagged by the SNPs examined here impact on both spherical equivalent and astigmatism via visual feedback.

The suggestive findings here that genes related to cell adhesion and synapse function may be involved in susceptibility to astigmatism is also consistent with the concept of genetic co-determination of spherical equivalent and refractive astigmatism, as several candidate genes identified in GWAS for spherical equivalent refractive error have putative roles in synapse function or plasticity, for example *RASGRF1*, *GRIA4*, *RBFOX1*, *LRRC4C*, *DLG2* (Kiefer et al. 2013; Verhoeven et al. 2013b; Stambolian et al. 2013; Hysi et al. 2010) as well as in cell adhesion, for example *TJP2*, *CTNND2*, *ANTXR2*, and *LRFN5* (Kiefer et al. 2013; Li et al. 2011; Verhoeven et al. 2013b).

### Comparison with previous work and limitations of the current study

Results from the meta-analysis of all cohorts for SNPs previously associated with astigmatism are shown in Table 4. Because the cohorts studied here overlap substantially with those examined previously (Fan et al. 2011; Lopes et al. 2013), low *P* values were expected—but not found. Thus the *P* values in Table 4 provide little evidence for replication of the previously associated markers. This is especially surprising for the corneal astigmatism-associated SNP at the *PDGFRA* locus (Fan et al. 2011), since this has already been replicated in a cohort of differing ethnicity (Guggenheim et al. 2013). Instead, the lack of replication may reflect the different traits examined (corneal versus refractive astigmatism). The other SNPs previously associated with astigmatism did not reach genome-wide significance in the original study, and were associated with astigmatism when analysed as a quantitative trait, which may explain the lack of independent replication.

**Table 4:** Results from the meta–analysis of all cohorts for SNPs previously associated with corneal astigmatism (CA) or refractive astigmatism (RA)

Trait	SNP	Chr	RA	NRA	RAF (min–max)	OR	95 % CI	*P* value	*I* ^2^	*N*	Gene	References
RA	rs3771395	2	A	G	0.06–0.30	1.04	1.00–1.09	5.17E−02	19.2	45,324	*VAX2*	Lopes et al. (2013)
CA	rs7677751	4	T	C	0.07–0.26	1.03	0.99–1.08	1.03E−01	17.9	45,287	*PDGFRA*	Fan et al. (2011)
RA	rs795544	5	C	A	0.64–0.92	1.05	1.01–1.09	2.01E−02	0	45,245	*DNAH5*	Lopes et al. (2013)
RA	rs10226930^a^										*SHH*	Lopes et al. (2013)
RA	rs485842	11	C	T	0.33–0.77	1.05	1.01–1.08	1.21E−02	10.2	45,137	*MAML2*	Lopes et al. (2013)
RA	rs12445126	16	A	G	0.02–0.14	1.02	0.97–1.09	4.16E−01	21.1	45,198	*XYLT1*	Lopes et al. (2013)
RA	rs11644988	16	G	A	0.73–0.99	1.04	0.98–1.11	2.46E−01	0	40,369	*FOXF1*	Lopes et al. (2013)

^a^SNP not present in current meta-analysis

Genetic studies of astigmatism are hampered by the variation in its magnitude and orientation with age, and its non-Gaussian frequency distribution, all of which complicate the choice of analysis model. In younger individuals, astigmatism is typically “with the rule” (WTR; axis of minus power cylindrical correcting lens close to horizontal) while in later life it usually switches to “against the rule” (ATR; correcting negative cylinder axis close to vertical) (Mandel et al. 2010; Guggenheim and Farbrother 2004). Amongst the theories explaining this transition, a loosening of eyelid tension is the most widely supported (Read et al. 2007). If it is the case that ATR and WTR astigmatism have different etiologies, then GWAS investigations should attain maximum statistical power by modelling younger and older subjects separately, modelling ATR and WTR astigmatism separately, or in modelling astigmatism as a vector quantity. However, the age-dependent shift in WTR to ATR largely concerns low-level astigmatism whereas higher levels may be more stable over the life course (Baldwin and Mills 1981; Weale 2003). Thus, the present study adopted a dichotomous case/control classification scheme, and analysed younger and older subjects separately, in an attempt to mitigate the effects of axis changes with age. The dichotomization scheme also allayed concerns regarding the non-normality of the trait, although this would have been at the expense of statistical power.

The use of a dichotomous phenotype definition for our GWAS meta-analysis of astigmatism contrasts with the quantitative trait approach used in previous GWAS meta-analyses by the CREAM consortium for refractive error and axial eye length (Verhoeven et al. 2013b; Cheng et al. 2013). It has been shown that binary trait GWAS meta-analysis results are sensitive to unequal numbers of cases and controls in individual cohorts, especially when the sample size is small (Willer et al. 2010). However, we found very similar results when overcoming this potential source of bias using an “effective sample size” rather than actual sample size during meta-analysis (Willer et al. 2010). In addition to the problem of unequal case/control sample sizes, we also observed highly inflated type-I errors during initial meta-analysis trials due to extreme OR estimates for a small number of low MAF markers in certain cohorts, e.g. if the minor allele was present in controls but absent in cases. To circumvent this, we pre-screened each GWAS results file, excluding markers with unfeasibly high OR estimates (OR < 0.2 or OR > 5.0).

Out of 7 Asian adult cohorts (total *N* = 9,295), 5 were Chinese cohorts (*N* = 5,132, about 55 % of the total Asian adult sample). Therefore, we cannot generalise our results from the Asian adult group with ease. Importantly, the SNP (rs7534824, in the gene *LOC101928334*) which showed the strongest suggestive association in the Asian group was only polymorphic in the Chinese cohorts (monomorphic in the Indian and Malay cohorts). For the other 3 SNPs reported in Table 2, although they are polymorphic in all three ethnic groups, the association signal was mainly driven by the observed association in the 5 Chinese cohorts.

In summary, this large-scale meta-analysis of GWAS studies for refractive astigmatism identified only a single locus that reached genome-wide significance (2p16.3, near *NRXN1*, in European adults) and there was no evidence for replication of this region in younger European individuals or in non-Europeans. Several putative candidate genes with functions relating to cell adhesion and/or synapse function were present in the next most strongly associated regions. Consistent with earlier work, all of the most strongly associated genetic variants identified had small effects, supporting the polygenic nature of genetic susceptibility to refractive astigmatism in the general population. Fewer candidate risk variants were discovered for refractive astigmatism than were found previously by the CREAM consortium for spherical equivalent refractive error (Verhoeven et al. 2013b), despite studying similar subject cohorts. Nevertheless, there was compelling evidence for shared genetic susceptibility for spherical and astigmatic refractive errors, implying that the co-occurrence of these traits is, at least in part, genetically determined.

### Electronic supplementary material

Below is the link to the electronic supplementary material.

Supplementary material 1 (PDF 914 kb)

## Appendix

The CREAM consortium Akira Meguro, Alan F. Wright, Alex W. Hewitt, Alvin L. Young, Amutha Barathi Veluchamy, Andres Metspalu, Andrew D. Paterson, Angela Döring, Anthony P. Khawaja, Barbara E. Klein, Beate St Pourcain, Ben A. Oostra, Brian Fleck, Caroline C. Klaver, Caroline Hayward, Cathy Williams, Cécile Delcourt, Cecilia Maubaret, Chi Pui Pang, Chiea-Chuen Khor, Ching-Yu Cheng, Christian Gieger, Christopher J. Hammond, Claire L. Simpson, Cornelia M. van Duijn, Daniel W. H. Ho, David A. Mackey, David M. Evans, Dwight Stambolian, Emily Chew, E-Shyong Tai, Evelin Mihailov, Federico Murgia, George Davey Smith, George McMahon, Ginevra Biino, Harry Campbell, Igor Rudan, Ilkka Seppala, Jaakko Kaprio, James F. Wilson, Jamie E. Craig, Jan Roelof Polling, Janina S. Ried, Jan-Willem Tideman, Jeremy A. Guggenheim, Jeremy R. Fondran, Jie Jin Wang, Jiemin Liao, Jing Hua Zhao, Jing Xie, Joan E. Bailey Wilson, John P. Kemp, Jost B. Jonas, Jugnoo S. Rahi, Juho Wedenoja, Kari-Matti Mäkelä, Kathryn P. Burdon, Kay-Tee Khaw, Kenji Yamashiro, Konrad Oexle, Laura Portas, Lindsay Farrer, Leslie J. Raffel, Li Jia Chen, Liang Xu, M. Kamran Ikram, Margaret M. Deangelis, Margaux Morrison, Maria Schache, Mario Pirastu, Mary-Frances Cotch, Masahiro Miyake, Maurice K.H. Yap, Maurizio Fossarello, Mika Kähönen, Mingguang He, Nagahisa Yoshimura, Nicholas G. Martin, Nicholas J. Timpson, Nick J. Wareham, Nobuhisa Mizuki, Norbert Pfeiffer, Olavi Pärssinen, Olli Raitakari, Ozren Polasek, Pancy O. Tam, Paul J. Foster, Paul Mitchell, Paul N. Baird, Peng Chen, Pirro G. Hysi, Puya Gharahkhani, Qiao Fan, René Höhn, Rhys D. Fogarty, Robert N. Luben, Robert P. Igo Jr, Robert Wojciechowski, Ronald Klein, S. Mohsen Hosseini, Sarayut Janmahasatian, Seang-Mei Saw, Seyhan Yazar, Shea Ping Yip, Sheng Feng, Songhomitra Panda-Jonas, Stuart MacGregor, Sudha K. Iyengar, Jonathan H. Lass, Taina Rantanen, Terho Lehtimäki, Terri L. Young, Thomas Meitinger, Tien Yin Wong, Tin Aung, Toomas Haller, Veronique Vitart, Vinay Nangia, Virginie J.M. Verhoeven, Vishal Jhanji, Wei Chen, Xiangtian Zhou, Xiaobo Guo, Xiaohui Li, Ya Xing Wang, Yi Lu, Yik-Ying Teo, Zoran Vatavuk.
