# Supplementary material for: Genome-wide association study for refractive astigmatism reveals genetic co-determination with spherical equivalent refractive error: the CREAM consortium
Source: Hum Genet. 2014 Nov 4;134(2):131–46. doi: 10.1007/s00439-014-1500-y (PMC4291519; doi:10.1007/s00439-014-1500-y)
Supplement: Supplementary file 1 — Supplementary material 1 (PDF 914 kb) [file 439_2014_1500_MOESM1_ESM.pdf]

Genome-Wide Association Study for Refractive Astigmatism Reveals Genetic Co-determination with Spherical Equivalent Refractive Error: The CREAM Consortium

Supplementary methods

Subjects, genotyping and association analysis in individual study cohorts

EUROPEAN ETHNICITY STUDIES

1958 British Birth Cohort

The 1958 British Birth Cohort (Rahi et al. 2011) is a prospective population-based cohort study that initially included 17,000 newborn children whose birth was within the first week of March 1958. All participants gave informed written consent to participate in genetic association studies, and the study was approved by the South East Multi Centre Research Ethics Committee (MREC) and the Oversight Committee for the biomedical examination of the British 1958 British birth cohort. Biomedical examination protocols were approved by the South East MREC. Assessment of refraction and astigmatism was undertaken in a random subsample of cohort members through non-cycloplegic autorefraction (Nikon Retinomax 2) of both eyes of each subject.

Illumina's Human1M-Duo chip was used for genotyping. Imputation was calculated with reference to HapMap release 22 CEU population data using IMPUTE version 2. Individuals were checked for genotyping success rate (all exceeded 99%), excess or low heterozygosity (all participating subjects were checked and found within the pre-defined interval of 0.2-04). SNPs were included in the analysis if they had a genotype success rate of at least 0.95, were within Hardy-Weinberg equilibrium ($p>10^{-04}$) and had a minor allele frequency of 0.04 or above. A logistic regression model was implemented in PLINK using sex and the first two principal components as covariates.

ALSPAC Mothers and Children (Avon Longitudinal Study of Parents and Children)

Details of this cohort study have been published previously (Boyd et al. 2013; Fraser et al. 2013). The research adhered to the tenets of the Declaration of Helsinki. Ethical approval for the study was obtained from the ALSPAC Law and Ethics committee and three local research ethics committees. Pregnant women with an expected date of delivery between 1st April 1991 and 31st December 1992, resident in the former Avon health authority area in Southwest England, were eligible to participate in this birth cohort study. 13,761 women were recruited. Data collection has been via various methods including self-completion questionnaires sent to the mother, to her partner and after age 5

to the child; direct assessments and interviews in a research clinic. As well as investigating the health and well-being of the children in the birth cohort, the health of the mothers is also an important area of investigation. For ALSPAC Mothers, DNA was extracted from blood samples collected as part of routine antenatal care, during attendance at ALSPAC research clinics, or from immortalized lymphoblastoid cell lines, for a total of 10,321 of the mothers. Non-cycloplegic autorefraction (Canon R50 instrument) was performed opportunistically when mothers accompanied their child to a research clinic visit, and/or by a researcher visiting their optician to obtain their spectacle prescription. Non-cycloplegic autorefraction data was used in preference to subjective refraction data when available. DNA samples were available for 11,343 ALSPAC Children, prepared from either blood samples or lymphoblastoid-transformed cell lines. Non-cycloplegic autorefraction (Canon R50 instrument) was performed during attendance at an ALSPAC research clinic visit when the children were approximately 15 years old. Genotyping was performed using Illumina 660 W-quad (Mothers) or Illumina HumanHap 550 (Children) bead arrays. Samples that did not cluster with HapMap CEU individuals on IBS plots, with excessive missingness (>5%), minimal or excessive autosomal heterozygosity, cryptic relatedness (>10% IBD) or with a sex-mismatch were excluded. SNPs with call rate <95%, minor allele frequency <1%, or Hardy-Weinberg P value < 10^{-7} were excluded. For the Mothers, genotypes were available for 8340 subjects, and there was a total of 1889 individuals with phenotype and genotype information available, and who passed all quality control filters (1402 with autorefraction data, 463 with subjective refraction data). For the children, there were 8365 subjects who had genotype information, and 3828 also had phenotype data and passed all quality control filters. Imputation was carried out separately for Mothers and Children. Markov Chain Haplotyping (MACH) v 1.0.16 was used to impute unobserved marker genotypes, with HapMap CEU build 36, release 22, genotypes as the reference set. Imputed SNP were required to have an imputation reliability score of RSQR >0.3. GWAS was performed using mach2qtl. Analysis was carried out separately for Mothers and Children, using mach2dat, with age and sex included as covariates. Analyses were run with and without the inclusion of the first 2 principal components: however, there was little evidence of population stratification (without PCs, λ_{GC}= 1.003 and 1.001 for Mothers and Children, respectively).

AREDS 1c

The Age-Related Eye Disease Study (AREDS) was initially designed as a long-term multicenter, prospective study of the clinical course of age-related macular degeneration (AMD) and age-related cataract (Age-Related Eye Disease Study Research 2001a, b). In addition to collecting natural history data, AREDS included a randomized clinical trial of high-dose vitamin and mineral supplements for AMD and a clinical trial of high-dose vitamin supplements for cataract (Age-Related Eye Disease

Study Research 2001a, b; Clemons et al. 2003). Prior to study initiation, the protocol was approved by an independent data and safety monitoring committee and by the institutional review board for each clinical center. Written informed consent was obtained from all participants before enrollment in accordance with the Declaration of Helsinki. AREDS participants were 55 to 80 years of age at enrollment and had to be free of any illness or condition that would make long-term follow-up or compliance with study medications unlikely or difficult. On the basis of fundus photographs graded by a central reading center, best-corrected visual acuity and ophthalmologic evaluations, 4,757 participants were enrolled in one of several AMD categories, including persons with no AMD (control group). Visual acuity measurement of all participants was performed with the standard procedure developed for the Early Treatment of Diabetic Retinopathy Study (ETDRS). A refraction measurement was performed for participants at the randomization visit and each annual visit. For those who experience a decrease of 10 letters from baseline visual acuity, refractions were also conducted at the non-annual visits. Blood samples were collected at baseline and longitudinally , and cell lines were established. DNA was extracted from cell lines according to standard protocols when the initial DNA supply has been depleted. For the current analysis, 1864 participants were included from the AREDS 1c population. Refractive error which was measured by a refraction protocol at baseline enrollment into the AREDS study (Age-Related Eye Disease Study Research 1999, 2001a, b; Clemons et al. 2003) was utilized for the definition of astigmatism. For AREDS 1c, genotyping of SNPs was performed using the Illumina HumanOmni2.5-4v1_B chip array and a genome-wide association study of astigmatism using the Illumina 2.5M chip was performed using a subset of the control group from the original AREDS study. These control individuals are all Caucasians, who do not have age-related macular degeneration (AMD) and were further screened to also exclude individuals with cataracts, retinitis pigmentosa or other retinal degenerations, color blindness, other congenital eye problems, LASIK, artificial lenses, and other eye surgery. For all studies, samples with low call rate (<98%), with low mean confidence scores over all non-missing genotypes, with chromosome anomalies, or with sex-mismatch were excluded. No samples exhibited excess heterozygosity rates (1.5 interquartile ranges above or below the upper/lower quartile ranges). Cryptic relatedness was detected by estimating IBD sharing and kinship coefficients among all possible pairs and one member of each pair exhibiting a sibling or closer relationship was dropped from the analysis. SNPs were dropped from the analysis if they exhibited more than 1 blind duplicate error, more than 1 HapMap control error or more than 1 error in HapMap control trios, a genotype call rate < 99%, minor allele frequency < 0.01, or Hardy-Weinberg P value < 10^{-4}. Tests for batch effects were not significant. No sex-specific differences in allelic frequency (>0.2) or heterozygosity (>0.3) were detected. Imputation was performed with the Markov Chain Haplotyping (MACH) package version 1.0.17 software (imputed to plus strand of NCBI build 36, HapMap release #22). For each imputed SNP, a reliability of imputation

was estimated as the ratio of the empirically observed dosage variance to the expected binomial dosage variance (O/E ratio). PLINK was used to perform the logistic regression association analyses, including age, sex and the first two principal components (to adjust for population stratification) as covariates. Genotype data from AREDS 1c are publicly available through the database of Genotype and Phenotype under the name of either the MMAP study or the AREDS study.

BATS and TEST Studies

The Australian Twin Eye Study comprises participants examined as part of the Twins Eye Study in Tasmania or the Brisbane Adolescent Twins Study. Details of the study are described elsewhere (Mackey et al. 2009). Ethical approval was obtained from the Royal Victorian Eye and Ear Hospital, the University of Tasmania, the Australian Twin Registry and the Queensland Institute of Medical Research. In all subjects post-cycloplegic (following instillation of tropicamide 1%) refraction for both eyes was measured using a Humphrey-598 automatic refractor (Carl Zeiss Meditec, Inc., Miami, Florida, USA). These measurements were used to determine the astigmatism trait analysed here.

DNA was extracted from blood leucocytes according to standard procedures. The Australian cohorts were genotyped on the Illumina Human Hap610 Quad array. SNPs with a genotype success rate of 0.95 or above was required for inclusion of the SNP into further steps of the analysis. Only SNPs in Hardy-Weinberg equilibrium were processed: the HWE inclusion threshold was $P>10x10^{-6}$. The minimum minor allele frequency required for inclusion of individual SNPs was 0.01. Imputation was calculated with reference to HapMap release 22 CEU using MACH (http://www.sph.umich.edu/csg/abecasis/MACH/).
Association analyses were performed using GeneABEL, adjusting for the sample relatedness and covariates such as age and sex. Ancestry for these individuals was determined initially through self-reporting and was verified through Principal Component decomposition of their ancestry with and without comparison with HapMap phase 2 European populations. Ancestral outliers were defined as having the first two principal components more than six standard deviations from the mean values of HapMap European samples, and therefore were subsequently excluded from the analyses.

CROATIA-Korčula Study

The CROATIA-Korčula study, Croatia, is a population-based, cross-sectional study that includes a total of 969 adult examinees, aged 18-98 (mean=56.3), from the Dalmatian island of *Korčula* and most (N=930) underwent a complete eye examination (Vitart et al. 2010). The study received approval from relevant ethics committees in Scotland and Croatia and followed the tenets of the Declaration of Helsinki. Non-cycloplegic autorefraction was measured on each eye using a NIDEK Ark30 hand-

held autorefractometer. Measures on eyes with a history of trauma, intra-ocular surgery, LASIK operations or keratoconus were removed. Analysis was performed as per analysis plan, excluding individuals with a cylinder power >= 5D in either eye and individuals with difference in cylinder power between right and left eyes beyond 4 standard deviations from the mean, and for over 25 year-old only as there were too few individuals in this study who were under 25 years of age. Genotypes were generated using a dense Illumina SNP array, 370CNV-Quad, following the manufacturer's standard recommendations. Genotypes were determined using the Illumina BeadStudio software. Samples with a call rate below 97 % , potentially mixed samples with excess autosomal heterozygosity or gender discrepancy (based on the sex chromosomes genotypes), and ethnic outliers (based on principal components analysis of genotypic data), were excluded from the analysis using the quality control algorithm implemented in the R package GenABEL. Imputation of allele dosage for over 2 millions SNPs on the 22 autosomal chromosomes with reference to HapMap CEU build 36 release 22 was performed using the software MACH v1.0.15 after exclusion of SNP with MAF < 0.01, call rate < 98% and HWE deviation $p< 10^{-6}$. After phenotypic and genotypic quality control steps, 826 individuals were available for the genetic association analysis. Genome-wide association analysis was performed using the ProbABEL package using an additive SNP allelic effect model and correcting for individual relatedness using the polygenic and mmscore functions implemented in the GenABEL package. Two ProbABEL analyses were run, one using the palogistic function which gives an estimate of OR and the second using the palinear function with the outcome as a quantitative trait and correcting for relatedness which yields corrected p-values of association upon which the standard errors of OR estimates are calculated.

CROATIA-Split Study

The CROATIA-Split study, Croatia, is a population-based, cross-sectional study in the Dalmatian City of Split that includes 1000 examinees aged 18-95. The study received approval from relevant ethics committees in Scotland and Croatia and followed the tenets of the Declaration of Helsinki. . Genotypes were generated using a dense Illumina SNP array, 370CNV-Quadv3, following the manufacturer's standard recommendations. Genotypes were determined using the Illumina BeadStudio software. Samples with a call rate below 97 % , potentially mixed samples with excess autosomal heterozygosity or gender discrepancy (based on the sex chromosomes genotypes), and ethnic outliers (based on principal components analysis of genotypic data), were excluded from the analysis using the quality control algorithm implemented in the R package GenABEL. Imputation of allele dosage for over 2 millions SNPs on the 22 autosomal chromosomes with reference to HapMap CEU build 36 release 22 was performed using the software MACH v1.0.15 after exclusion of SNP with

MAF < 0.01, call rate < 98% and HWE deviation p< 10^{-} 343 individuals with good quality genotypes were used in this analysis. Traits analysed and analyses were as in the CROATIA-Korcula Study

CROATIA-Vis Study

The CROATIA-Vis study, Croatia, is a population-based, cross-sectional study including adult participants, aged 18–93 years (mean = 56), from the Dalmatian island of Vis, a subset of which (N=640) underwent a complete eye examination in summer 2007 and provided their ophthalmologic history (Vitart et al. 2010). The study received approval from relevant ethics committees in Scotland and Croatia and followed the tenets of the Declaration of Helsinki. Genotypes were generated using a dense Illumina SNP array, HumanHap 300v1, following the manufacturer's standard recommendations. Genotypes were determined using the Illumina BeadStudio software. Samples with a call rate below 97 % , potentially mixed samples with excess autosomal heterozygosity or gender discrepancy (based on the sex chromosomes genotypes), and ethnic outliers (based on principal components analysis of genotypic data), were excluded from the analysis using the quality control algorithm implemented in the R package GenABEL. Imputation of allele dosage for over 2 millions SNPs on the 22 autosomal chromosomes with reference to HapMap CEU build 36 release 22 was performed using the software MACH v1.0.15 after exclusion of SNP with MAF < 0.01, call rate < 98% and HWE deviation p< 10^{-6}. After phenotypic and genotypic quality control steps, 529 individuals were available for the genetic association analysis Traits analysed and analyses were as in the CROATIA-Korcula Study

Erasmus Rucphen Family Study (ERF)

The Erasmus Rucphen Family (ERF) Study is a family-based cohort in a genetically isolated population in the southwest of the Netherlands with over 3,000 participants aged between 18 and 86 years. Cross-sectional examination took place between 2002 and 2005. The rationale and study design of this study have been described elsewhere (Aulchenko et al. 2004; Pardo et al. 2005). Cross-sectional examination took place between 2002 and 2005, including a non-dilated automated measurement of refractive error using a Topcon RM-A2000 autorefractor. All measurements in these studies were conducted after the Medical Ethics Committee of the Erasmus University had approved the study protocols and all participants had given a written informed consent in accordance with the Declaration of Helsinki.

DNA was genotyped on one of four different platforms (Illumina 6k, Illumina 318K, Illumina 370K and Affymetrix 250K). Samples with low call rate (<97.5%), with excess autosomal heterozygosity (>0.336), or with sex-mismatch were excluded, as were outliers identified by the identity-by-state

clustering analysis (outliers were defined as being >3 s.d. from population mean or having identity-by-state probabilities >97%). A set of genotyped input SNPs with call rate >98%, with minor allele frequency >0.01, and with Hardy-Weinberg P value $>10^{-6}$ was used for imputation. We used the Markov Chain Haplotyping (MACH) package version 1.0.15 software (Rotterdam, The Netherlands; imputed to plus strand of NCBI build 36, HapMap release #22) for the analyses. For each imputed SNP, a reliability of imputation was estimated as the ratio of the empirically observed dosage variance to the expected binomial dosage variance (O/E ratio). GWAS analyses were performed using the ProbABEL package. Mmscore models were used to correct for family structure. We used age and sex as covariates.

Finnish Twin Study on Aging (FITSA)

Finnish Twin Study on Aging (FITSA) (Parssinen et al. 2010) is a study of genetic and environmental effects on the disablement process in older female twins. The FITSA participants were 103 MZ and 114 DZ Finnish twin pairs (424 individuals, all Caucasian women) aged 63-76 years who took part in multiple laboratory examination in 2000, 2003 and responded in questionnaires in 2011. Before the examinations, the subjects provided a written informed consent according to the Declaration of Helsinki. The study protocol was approved by the ethics committee of the Central Hospital District of Central Finland.

DNA was extracted from EDTA-anticoagulated whole blood according to standard procedures. Because the genotyping was part of a larger project, the GenomEUtwin project, three different genotyping platforms, the MegaBACE1000 (Amersham Biosciences) electrophoresis system, the ABI3700, and the ABI3730 (Applied Biosystems) automated electrophoresis systems were used. The genotype calls were made with the GeneticProfiler1.5 (MegaBACE1000) and GeneMapper3.7 (ABI3700 and ABI3730) software. The genotyping quality control thresholds included minor allele frequency >0.01, success rate by marker >0.95, success rate by individual >0.95, and HWE P>0.000001. Oxford Impute2 program was used to generate the Hapmap2 CEU release 24 imputed SNPs. The posterior probability threshold for "best-guess" imputed genotypes was 0.9. Logistic regression analyses of astigmatism were performed using Plink 1.07 with age included in the model as a covariate.

Framingham Eye Study

The Framingham Eye Study (Leibowitz et al. 1980) (FES) was nested within the Framingham Heart Study (FHS, http://www.framinghamheartstudy.org), which began its first round of extensive physical examinations in 1948 by recruiting 5,209 men and women from the town of Framingham, MA, USA.

Surviving participants from the original cohort returned for biennial exams, which continue to the present. A total of 2675 FHS participants were also examined as part of the FES between 1973 and 1975. The FES was designed to evaluate ocular characteristics of examinees such as: senile cataract; age-related macular disease; glaucoma; and retinopathy. Between 1989 and 1991, 1603 offspring of original cohort participants also received ocular examinations (Framingham Eye Study Group 1996). The analyses in the current study are limited to 1532 participants (43.9% men) from both the original and the offspring cohorts for whom both phenotype and genotype data were available. Most individuals in this analysis set are unrelated but a small number of related pairs remain. All data--including refractive error, demographics and genotypes--were retrieved from the database of Genotypes and Phenotypes (dbGaP, http://www.ncbi.nlm.nih.gov/gap) after approval for controlled access to individual-level data. All study protocols are in compliance with the World Medical Association Declaration of Helsinki. Since 1971, written consent has been obtained from participants before each examination. The research protocols of the Framingham Heart Study are reviewed annually by the Institutional Review Board of the Boston University Medical Center and by the Observational Studies Monitoring Board of the National Heart, Lung and Blood Institute.

Genotyping was conducted as part of the NHLBI Framingham SNP Health Association Resource (SHARe). This sub-study contains genotype data for approximately 550000 SNPs (Affymetrix 500K mapping arrays [Mapping250k_Nsp and Mapping250K_Sty] plus Affymetrix 50K supplemental human gene-focused array) in over 9200 FHS participants (1497 of whom were used in this analysis). Samples were chosen based on pedigree information and genotyping quality; samples with a genotypic call rate below 95% were not chosen for analysis. The mean call rate for analyzed samples was 99.2% (SD=0.4%). Genotype data cleaning was carried-out in several steps. The final marker list contained 436,494 high-quality SNPs with a minor-allele frequency >= 0.01, a Mendelian error rate below 2% across all pedigrees, a genotype call rate above 95%, and whose distribution was consistent with Hardy-Weinberg expectations (P>0.0001). Genotype imputation to the HapMap-II reference panel (CEU population release 22, NCBI build 36) was carried out in a two-step process using the Markov Chain Haplotyping (MACH version 1.0.16.a) software. First, crossover and error-rate maps were built using 400 unrelated individuals (200 male and 200 female) sampled from FHS subjects. Second, genotype imputations of approximately 2.5 million autosomal HapMap-II SNPs were carried out on the entire FHS dataset using parameters estimated from step 1. Logistic regression analyses of astigmatism were performed using PLINK with age, sex and the first two principal components (to adjust for population stratification) included in the model as covariates.

Gutenberg Health Study (GHS1, GHS2)

The Gutenberg Health Study (GHS) is a population-based, prospective, observational cohort study in the Rhine-Main Region in midwestern Germany with a total of 15,000 participants and follow-up after five years. The study sample is recruited from subjects aged between 35 and 74 years at the time of the exam. The sample was drawn randomly from local governmental registry offices and stratified by gender, residence (urban and rural) and decade of age. Exclusion criteria were insufficient knowledge of the German language to understand explanations and instructions, and physical or psychic inability to participate in the examinations in the study center. Individuals were invited for a 5-hour baseline-examination to the study center where clinical examinations and collection of blood samples were performed. An important feature of the study design is the interdisciplinary combination of an ophthalmological examination, general and especially cardiovascular examinations, psychosomatic evaluation, laboratory tests, and biobanking for proteomic and genetic analyses. All participants underwent an ophthalmological investigation of 25 minutes' duration taking place between 11:00 a.m. and 8:00 p.m. This examination was based on standard operating procedures and included a medical history of eye diseases, autorefraction and visual acuity testing (Humphrey® Automated Refractor/Keratometer (HARK) 599™, Carl Zeiss Meditec AG, Jena, Germany), visual field screening using frequency doubling technology (Humphrey® Matrix Perimeter, Carl Zeiss Meditec AG, Jena, Germany), central corneal thickness and keratometry measurement (Scheimpflug imaging with the Pachycam™, Oculus, Wetzlar, Germany), IOP measurement with a non-contact tonometer (Nidek NT-2000™, Nidek Co., Japan), slitlamp biomicroscopy with undilated pupils (Haag-Streit BM 900®, Bern, Switzerland) and non-mydriatic fundus photography (Visucam PRO NM,™, Carl Zeiss Meditec AG, Jena, Germany), all administered by an ophthalmologist. The study was approved by the Medical Ethics Committee of the University Medical Center Mainz and by the local and federal data safety commissioners. According to the tenets of the Declaration of Helsinki, written informed consent was obtained from all participants prior to entering the study.

Within GHS, DNA was extracted from buffy-coats from EDTA blood samples as described in Zeller *et al.*(Zeller et al. 2010). Genetic analysis was conducted in the first 5,000 study participants. For these, 3,463 individuals were genotyped in 2008 (GHS1) and further 1,439 individuals in 2009 (GHS2). Genotyping was performed for GHS1 and GHS2 using the Affymetrix Genome-Wide Human SNP Array 6.0 (http://www.affymetrix.com), as described by the Affymetrix user manual. Genotypes were called using the Affymetrix Birdseed-V2 calling algorithm. Individuals with a call rate below 97% or a too high autosomal heterozygosity (3 s.d. from mean) and sex-mismatches were excluded. After applying standard quality criteria (minor allele frequency >1%, genotype call rate >98% and P-value

of deviation from Hardy-Weinberg equilibrium of >0.0001), 675,350 SNPs in 2791 individuals from GHS1 and 673,914 SNPs in 1,163 individuals from GHS2 remained for analysis (total 3954). Imputation of missing genotypes was performed using Impute software v2.1.0 and HapMap release 24, NCBI Build 36. Logistic regression analyses of astigmatism were performed using SNPTEST with age, sex and the first two principal components (to adjust for population stratification) included in the model as covariates.

KORA

KORA ("Kooperative Gesundheitsforschung in der Region Augsburg" which translates as "Cooperative Health Research in the Region of Augsburg") is a population based study of adults randomly selected from 430,000 inhabitants living in Augsburg and 16 surrounding counties in Germany (Holle et al. 2005; Oexle et al. 2011; Steffens et al. 2006; Wichmann et al. 2005). The collection was done in 4 separate groups from 1984-2001 (S1-S4). All survey participants are residents of German nationality identified through the registration office. In the KORA S3 and S4 studies 4,856 and 4,261 subjects have been examined implying response rates of 75% and 67%, respectively. 3,006 subjects participated in a 10-year follow-up examination of S3 in 2004/05 (KORA F3), and 3080 of S4 in 2006/2008 (KORA F4). The age range of the participants was 25 to 74 years at recruitment. The study was approved by the local ethics committee. Written informed consent was obtained from all participants before enrollment in accordance with the Declaration of Helsinki. Genome-wide genotyping using the Illumina 2.5M chip was performed on a subset of 1981 individuals in the S3/F3 (mean age 55.7, range 35–84) who had measurements of refractive error and available DNA samples. For each subject, eyeglass prescriptions were measured in addition to an evaluation using the Nikon Retinomax. The individuals included in this GWAS are all Caucasian, do not have age-related macular degeneration, cataracts, retinitis pigmentosa, color blindness, other congenital eye problems, LASIK, artificial lenses, and other eye surgery. DNA was extracted from cell lines according to standard protocols. Genotyping of SNPs was performed using the Illumina HumanOmni2.5-4v1_B chip array.). Samples with low call rate (<98%), with low mean confidence scores over all non-missing genotypes, with chromosome anomalies, or with sex-mismatch were excluded. No samples exhibited excess heterozygosity rates (1.5 interquartile ranges above or below the upper/lower quartile ranges). Cryptic relatedness was detected by estimating IBD sharing and kinship coefficients among all possible pairs and one member of each pair that exhibited a sibling or closer relationship was dropped from the analysis. SNPs were dropped from the analysis if they exhibited more than 1 blind duplicate error, more than 1 HapMap control error or more than 1 error in HapMap control trios, a genotype call rate < 99%, minor allele frequency < 0.01, or Hardy-Weinberg *P*-value < 10^{-4}. Tests for batch effects were not significant. No sex-specific differences in allelic frequency (>0.2) or

heterozygosity (>0.3) were detected. Eigenstrat did not detect significant population stratification and the genomic control inflation factor was 1.014. A subset of the retained SNPs was used for imputation with the Markov Chain Haplotyping (MACH) package version 1.0.17 software (imputed to plus strand of NCBI build 36, HapMap release #22). For each imputed SNP, a reliability of imputation was estimated as the ratio of the empirically observed dosage variance to the expected binomial dosage variance (O/E ratio). Logistic regression using PLINK (http://pngu.mgh.harvard.edu/purcell/plink) was used to perform the association analyses of astigmatism with age and sex included as covariates.

Ogliastra Genetic Park, Talana study (OGP Talana)

A cross-sectional ophthalmic study was performed in Talana, Perdasdefogu and Urzulei within the Ogliastra Project, a large epidemiological survey conducted in a geographically, culturally and genetically isolated population living in an eastern-central region of Sardinia (Biino et al. 2005). In Talana the study was carried out between October 2001 and October 2002 and adhered to the tenets of the declaration of Helsinki. Talana is an Ogliastran village situated at an altitude of 700 m above sea level in one of the most secluded areas of Sardinia; it has about 1200 inhabitants and, importantly, archival records are available from 1589 and genealogical trees have been reconstructed from 1640. 789 volunteers gave their written informed consent and were invited to the local medical centre, which was equipped with a complete set of ophthalmic instruments for this survey. All participants underwent a complete eye examination conducted according to a standardized protocol that included visual acuity measurement with Snellen charts at a distance of 5 m, autorefraction (RK-8100 Topcon, Tokyo, Japan) assessing sphere, cylinder and axis, slit lamp biomicroscopy (Model BQ900, Haag-Streit, Bern, Switzerland), contact tonometry and colour fundus photography (TRC-50IA,Topcon) and non-contact optical biometry (IOLMaster,Carl Zeiss, Italy) and Optical coherence tomography (OCT). Whole blood was obtained from all consenting family members of Talana village for DNA extraction. Genotyping was carried out using the Affymetrix 500k chips using standard protocols. SNPs quality control was performed using the GenABEL software package in R. Samples with overall SNP call rate < 93%, with minor allele frequency < 0.01, and with Hardy-Weinberg P value $>10^{-6}$, showing excess of heterozygosity, or being classified as outliers by allelic identity-by-state (IBS) clustering analysis, were excluded. Using the phase II CEU HapMap individuals (release 22, NCBI build 36) as reference panel for imputation, we imputed genotypes to nearly 2.5 milion SNPs using MACH. SNPs imputed with Rsq <0.3 were excluded. Genome-wide association analysis was performed using an additive SNP allelic effect model and correcting for individual relatedness using the polygenic and mmscore functions as implemented in the GenABEL package. Sex and age were used as covariates.

Orkney Complex Disease Study (ORCADES)

The Orkney Complex Disease Study (ORCADES) is a population-based, cross-sectional study in the Scottish archipelago of Orkney, including 1,285 individuals with eye measurements. The study received approval from relevant ethics committees in Scotland and followed the tenets of the Declaration of Helsinki. Autorefractive measurements were obtained using a Kowa KW 2000 autorefractometer. Measures on eyes with a history of trauma, intra-ocular surgery, LASIK operations or keratoconus were removed. Analysis was performed as per analysis plan excluding individuals with a cylinder power >= 5D in either eye and individuals with difference in cylinder power between right and left eyes beyond 4 standard deviations from the mean, and for over 25 year-old only as under 25 year were too few. . Genotypes were generated using a dense Illumina SNP arrays, HumanHap 300v2 and 370CNV-Quad, following the manufacturer's standard recommendations. Genotypes were determined using the Illumina BeadStudio software. Samples with a call rate below 97 % , potentially mixed samples with excess autosomal heterozygosity or gender discrepancy (based on the sex chromosomes genotypes), and ethnic outliers (based on principal components analysis of genotypic data), were excluded from the analysis using the quality control algorithm implemented in the R package GenABEL. Imputation of allele dosage for over 2 millions SNPs on the 22 autosomal chromosomes with reference to HapMap CEU build 36 release 22 was performed using the software MACH v1.0.15 after exclusion of SNP with MAF < 0.01, call rate < 98% and HWE deviation $p < 10^{-6}$. 502 individuals which had been genotyped and passed genotyping quality control were used in this analysis. Genome-wide association analysis was performed using the ProbABEL package using an additive SNP allelic effect model and correcting for individual relatedness using the polygenic and mmscore functions implemented in the GenABEL package. Two ProbABEL analyses were run, one using the palogistic function which gives an estimate of OR and the second using the palinear function with the outcome as a quantitative trait and correcting for relatedness which yields corrected p-values of association upon which the standard errors of OR estimates are calculated.

RAINE Eye Health Study (REHS)

The Raine Eye Health Study (REHS) was conceived to determine the prevalence of and risk factors for eye disease in young adults, and to characterize ocular biometric parameters in a young adult cohort (Yazar et al. 2013). The Western Australian Pregnancy Cohort (Raine) Study originated as a randomized-controlled trial of 2900 women recruited from the state's largest maternity hospital. Their offspring (N=2868) have been followed at birth, ages 1, 2, 3, 5, 8, 10, 14, 17 and 20 years of age in a prospective cohort study. DNA was collected from participants for genome-wide association studies and genotyping was performed using Illumina 660 Quad Array. Any pair of individuals who

were related with a π > 0.1875 (in between second and third degree relatives – e.g. between half-sibs and cousins) was investigated, and the individual with the higher proportion of missing data was excluded from the 'clean' dataset (68 individuals excluded). Individuals who had low genotyping success (i.e. missing data) were excluded from the 'clean' dataset – a threshold of absent data > 3% was used for exclusion (16 individuals excluded). Additionally, if they had high levels of heterozygosity then they were also excluded (heterozygosity < 0.30 excluded 3 individuals). SNPs which did not satisfy a Hardy-Weinburg equilibrium p-value > 5.7x10-7 (919 markers), a call rate >95% (97,718 markers), and a minor allele frequency >0.01 (1%) (119,246 markers – includes CNV's) were excluded. To account for population stratification, the first five principal components were calculated using a subset of 42,888 SNPS that were not in LD with each other. Principal component analysis was conducted using the EIGENSTRAT program. The MACH v1.0.16 (http://www.sph.umich.edu/csg/yli/mach/index.html) software was used for GWAS imputation on the 22 autosomes. Once the data were cleaned, a two step process was carried out using the CEU samples from HapMap phase2 build 36 release 22 (http://hapmap.ncbi.nlm.nih.gov/index.html.en) as a reference panel. At the 20-year follow-up participants completed a comprehensive eye assessment that included visual acuity, orthoptic assessment and cycloplegic autorefraction, as well as several ocular biometric variables and multiple ophthalmic photographs of the anterior and posterior segments. Using the 20 year follow-up examination refractive error phenotypes, 1007 Caucasian participants with both astigmatism case/control phenotypes and high quality genotypes were included in the current analysis. Logistic regression using R with a Plink interface was used to perform the association analyses of astigmatism with age, sex and the first two principal components included as covariates.

Rotterdam Study (RS1, RS2, RS3)

The Rotterdam Study is a prospective population-based cohort study in the elderly living in Ommoord, a suburb of Rotterdam, the Netherlands. Details of the study are described elsewhere (Hofman et al. 2011). In brief, the Rotterdam Study consists of 3 independent cohorts: RS1, RS2, and RS3. For the current analysis, 5,422residents aged 55 years and older were included from RS1, 1,973 participants aged 55 and older from RS2, and 1,971 aged 45 and older from RS 3. 99% of subjects were of Caucasian ancestry. Participants underwent multiple physical examinations with regular intervals from 1991 to present, including a non-dilated automated measurement of refractive error using a Topcon RM-A2000 autorefractor. All measurements in RS-1–3 were conducted after the Medical Ethics Committee of the Erasmus University had approved the study protocols and all participants had given a written informed consent in accordance with the Declaration of Helsinki. DNA was extracted from blood leucocytes according to standard procedures. Genotyping of SNPs

was performed using the Illumina Infinium II HumanHap550 chip v3.0 array (RS-I); the HumanHap550 Duo Arrays and the Illumina Human610-Quad Arrays (RS-II), and the Human 610 Quad Arrays Illumina (RS-III). Samples with low call rate (<97.5%), with excess autosomal heterozygosity (>0.336), or with sex-mismatch were excluded, as were outliers identified by the identity-by-state clustering analysis (outliers were defined as being >3 s.d. from population mean or having identity-by-state probabilities >97%). We used genomic control to obtain optimal and unbiased results and applied the inverse variance method of each effect size estimated for both autosomal SNPs that were genotyped and imputed in both cohorts. A set of genotyped input SNPs with call rate >98%, with minor allele frequency >0.01, and with Hardy-Weinberg P value $>10^{-6}$ was used for imputation. We used the Markov Chain Haplotyping (MACH) package version 1.0.15 software (Rotterdam, The Netherlands; imputed to plus strand of NCBI build 36, HapMap release #22) for the analyses. For each imputed SNP, a reliability of imputation was estimated as the ratio of the empirically observed dosage variance to the expected binomial dosage variance (O/E ratio). GWAS analyses were performed using GRIMP with age and sex included as covariates.

TwinsUK

The TwinsUK adult twin registry based at St. Thomas' Hospital in London is a volunteer cohort of over 10,000 twins from the general population (Spector and Williams 2006). Twins largely volunteered unaware of the eye studies, gave fully informed consent under a protocol reviewed by the St. Thomas' Hospital Local Research Ethics Committee and underwent non-cyclopleged autorefraction using an ARM-10 autorefractor (Takagi Ltd). Out of the original 4,388 subjects for whom phenotype and genotype information was available, 2658 subjects were included in the current analysis. Genotyping was carried out using three genotyping platforms from Illumina: the HumanHap 300k Duo for part of the UK Twin Cohort and the HumanHap610-Quad array for the rest of the UK Twin Cohort. Imputation was calculated with reference to HapMap release 22 CEU population data using IMPUTE version 2. Individuals were included if their genotyping success rate exceeded 95%, did not show excess or low heterozygosity (defined by the interval interval of 0.2-04). SNPs were included in the imputation if they had a genotype success rate of at least 0.95 if their minor allele frequency was superior to 0.005 and at least 0.99 if their MAF was 0.01-0.05. Only SNPs that were within Hardy-Weinberg equilibrium ($p>10^{-04}$) and had a minor allele frequency of 0.04 or above were regressed. Logistic regression analyses of astigmatism were performed using PLINK with age, sex and the first two principal components (to adjust for population stratification) included in the model as covariates.

Wisconsin Epidemiologic Study of Diabetic Retinopathy (WESDR)

WESDR is an observational cohort study of diabetes complications (1979-2007)(Klein et al. 2010). Subjective refraction, measured following standard protocols at first visit, was analyzed in the current study (n=589).

Subjects with type 1 diabetes from WESDR were genotyped using Illumina HumanOmni1-Quad BeadChip assay. Individuals showing gender mismatch with typed X-linked markers (n=8), cryptic relatedness (n=5), high autosomal heterozygosity (n=6), call rate <0.95 (n=30), as well as ethnicities other than "white" were not included in the analysis. Population genetic approaches based on multi-dimensional scaling implemented in PLINK v1.07 were used to identify and exclude ethnically admixed individuals. Imputation was performed in IMPUTE v2.2.2 using all populations from HapMap phase II release 22 as reference (IMPUTE2 chooses the best custom reference set for each individual internally). Association analysis was performed by PLINK v1.07 using genotype dosages to account for imputation uncertainty. The regression model accounted for age, gender and the first two principal components.

Young Finns Study (YFS)

The YFS cohort is a Finnish longitudinal population study sample on the evolution of cardiovascular risk factors from childhood to adulthood (Raitakari et al. 2008). The first cross-sectional study was conducted in the year 1980 in five different centers. It included 3,596 participants in the age groups of 3, 6, 9, 12, 15, and 18, who were randomly chosen from the national population register. After the baseline in 1980 these subjects have been re-examined in 1983 and 1986 as young individuals, and in 2001, 2007 and 2011 as older individuals. For the current analysis a subsample from the newest (2011) follow-up was used from four centers (N=1480) where the refractive error measurements data from both eyes were available.

This study was carried out in accordance with the recommendations of the Declaration of Helsinki. All participants provided written informed consent and the study protocol was approved by the Ethics Committee.

Genomic DNA was extracted from peripheral blood leukocytes using a commercially available kit and Qiagen BioRobot M48 Workstation according to the manufacturer's instructions (Qiagen, Hilden, Germany). Genotyping was done for 2,556 samples using custom build Illumina Human 670k BeadChip at Welcome Trust Sanger Institute. Genotypes were called using Illuminus clustering algorithm. 56 samples failed Sanger genotyping pipeline QC criteria (i.e., duplicated samples, heterozygosity, low call rate, or Sequenom fingerprint discrepancy). From the remaining 2,500 samples one sample failed gender check, three was removed due to low genotyping call rate (< 0.95)

and 54 samples for possible relatedness (pi-hat > 0.2) . 11,766 SNPs were excluded based on Hardy–Weinberg equilibrium (HWE) test ($p \leq 10^{-6}$), 7,746 SNPs failed missingness test (call rate < 0.95) and 34,596 SNPs failed frequency test (MAF < 0.01). After quality control there were 2,442 samples and 546,677 genotyped SNPs available for further analysis (Smith et al. 2010). Genotype imputation was performed using MACH (Booij et al. 2011; Li et al. 2009) 1.0 and HapMap II CEU (release 22, NCBI build 36, dbSNP 126) samples as reference. Palindromic A/T and C/G SNPs were removed before imputation. After imputation there were 2,543,887 SNPs available. SNPs with squared correlation between imputed and true genotypes ≥ 0.30 were considered well imputed. Logistic regression analyses of astigmatism were performed using SNPTEST v.2.1.1.with age, sex and the first two principal components (to adjust for population stratification) included in the model as covariates.

ASIAN ETHNICITY STUDIES

Beijing Eye Study (BES)

The BES is a population-based cohort of Han Chinese in the rural region and in the urban region of Beijing in North China. The Medical Ethics Committee of the Beijing Tongren Hospital approved the study protocol and all participants gave informed consent, according to the Declaration of Helsinki. At baseline (2001), 4439 individuals out of 5324 eligible individuals aged 40 years or older participated (response rate: 83.4%). In the years 2006 and 2011, the study was repeated by re-inviting all participants from the survey from 2001 to be re-examined. Out of the 4439 subjects examined in 2001, 3251 (73.2%) subjects returned for the follow-up examination in 2006, and 2695 (60.7%) subjects returned for the follow-up examination in 2011. For all subjects, visual acuity was measured. Automatic refractometry (Auto Refractometer AR-610, Nidek Co., Ltd, Tokyo, Japan) was performed if uncorrected visual acuity was lower than 1.0. The values obtained by automatic refractometry were verified and refined by subjective refractometry. Refraction data collected in 2011 was used in the analysis. In the survey of 2006, blood samples were taken from 2,929 (90.1%), and DNA was extracted from blood leucocytes according to standard procedures. We performed genotyping using Illumina Human610-Quad BeadChip in 988 subjects (Cornes et al. 2012). Of them, we excluded 151 with cryptic relatedness during sample QC procedure. Additional 259 Individuals with cataract surgery or missing refraction data were also excluded. This left a total of 585 individuals for analysis. Logistic regression analyses of astigmatism were performed using 585 individuals with age, sex and the first two principal components (to adjust for population stratification) included in the model as covariates.

Singapore Prospective Study Program (SP2)

Samples of SP2 were from a revisit of two previously conducted population-based surveys carried out in Singapore between 1992 and 1998, including the National Health Survey 1992 and the National Health Survey 1998(Hughes et al. 1997). These studies comprise random samplings of individuals stratified by ethnicity from the entire Singapore population. A total of 8266 subjects were invited in this follow-up survey and 6301 (76.1% response rate) subjects completed the questionnaire, of which 4056 (64.4% of those who completed the questionnaire) also attended the health examination and donated blood specimens. The present GWA genotyping for SP2 involved individuals of Chinese descent only (n=2,867)(Sim et al. 2011).

Of the 2,867 blood-derived DNA samples, 392 samples were genotyped on the HumanHap 550v3, 1,459 samples on the 610-Quad, 817 samples on the 1M-Duov3, 191 samples on both 550v3 and 1M-Duov3, and 8 samples on both 610-Quad and 1M-Duov3. For the samples that were genotyped on two platforms, we used the genotypes from the denser platform in our study. We excluded 443 individuals on the following conditions, sample call rates of less than 95%, excessive heterozygosity, cryptic relatedness by IBS, population structure ascertainment, and gender discrepancies as listed in the main text. This left 2,434 post-QC SP2 samples. During the SNPs QC procedure, we excluded SNPs with low genotyping call rates (> 5% missingness) or monomorphic, with MAF < 1%, or with significant deviation from HWE ($P < 10^{-6}$). This yielded a post-QC set of 462,580 SNPs. As SP2 samples are genotyped on different platforms, the concordance of the duplicate samples plated on different Beadarrays chips was also examined as quality of genotyping. The average SNP concordance rate between chips for the post-QC duplicated samples was 0.995. We additionally assessed the SNPs that are present on different platforms for extreme variations in allele frequencies with a 2-degree of freedom chi-square test of proportions, removing 62 SNPs with *P*-values < 0.0001.
A total of 1954 individuals had both high quality genotype data and astigmatism trait data and were used in the present logistic regression analysis, with age and sex included in the model as covariates.

Singapore Malay Eye Study (SiMES)

SiMES is a population-based prevalence survey of Malay adults aged 40 to 79 years living in Singapore that was conducted between August of 2004 and June of 2006(Foong et al. 2007). From a Ministry of Home Affairs random sample of 16,069 Malay adults in the Southwestern area, an age-stratified random sampling strategy was used in selecting 1400 from each decade from age 40 years onward (40–49, 50–59, 60–69, and 70–79 years).The 4,168 eligible participants from the sampling frame, while 3280 (78.7%) participated. Genome-wide genotyping was performed in 3,072

individuals (Cornes et al. 2012; Vithana et al. 2011).
Total of 3,072 DNA samples were genotyped using the Illumina Human 610 Quad Beadchips (Khor et al. 2011; Vithana et al. 2011). Using the same quality control criteria, we omitted a total of 530 individuals including those of subpopulation structure (n=170), cryptic relatedness (n=279), excessive heterozygosity or high missingness rate > 5% (n=37), and gender discrepancy (n=44). A total of 2165 individuals were over age 25 and had high quality genotypes and phenotypes for astigmatism. After the removal of the samples, SNP QC was then applied on a total of 579,999 autosomal SNPs for the 2,542 post-QC samples. SNPs were excluded based on (i) high rates of missingness (> 5%) ; (ii) monomorphism or MAF < 1% ; or (iii) genotype frequencies deviated from HWE ($p < 1 \times 10^{-6}$). Logistic regression analyses of astigmatism were performed using 2165 individuals with age, sex and the first two principal components (to adjust for population stratification) included in the model as covariates.

Singapore Indian Eye Study (SINDI)

SINDI is a population-based survey of major eye diseases (Lavanya et al. 2009) in ethnic Indians aged 40 to 80 years living in the South-Western part of Singapore and was conducted from August 2007 to December 2009. In brief, 4,497 Indian adults were eligible and 3,400 participated. Genome-wide genotyping was performed in 2,953 individuals (Khor et al. 2011). Participants were excluded from the study if they had cataract surgery and missing refraction data.
The Illumina Human610 Quad Beadchips was used for genotyping all DNA samples from SINDI (n=2,593). We excluded 415 subjects from the total of 2,953 genotyped samples based on: excessive heterozygosity or high missingness rate > 5% (n=34) , cryptic relatedness (n=326), issues with population structure ascertainment (n=39) and gender discrepancies (n=16). This left a total of 2,538 individuals with 579,999 autosomal SNPs and 1998 of these individuals were also over age 25 and had astigmatism phenotype data. During SNP QC procedure. SNPs were excluded based on (i) high rates of missingness (> 5%) ; (ii) monomorphism or MAF < 1% ; or (iii) genotype frequencies deviated from HWE ($p < 1 \times 10^{-6}$). Logistic regression analyses of astigmatism were performed using 1998 individuals with age, sex and the first two principal components (to adjust for population stratification) included in the model as covariates.

Singapore Chinese Eye Study (SCES)

Similar to SINDI, the Singapore Chinese Eye Study (SCES) is a population-based cross-sectional study of eye diseases in Chinese adults 40 years of age or older residing in the southwestern part of Singapore. The methodology of the SCES study has been described in detail previously. Between 2009 and 2011, 3,353 (72.8%) of 4,605 eligible individuals underwent a comprehensive

ophthalmologic examination, using the same protocol as SINDI (Foong et al. 2007). Genome-wide genotyping using was done in a subset of 1,952 SCES participants using Illumina Human610-Quad BeadChip (Cornes et al. 2012) The same QC methods used for SiMES and SINDI were applied to the SCES genotyping samples: samples were excluded if they showed evidence of admixture, cryptic relatedness, high heterogeneity and gender discrepancies. From a starting number of 1,952 individuals, three samples had per-sample call rate of <95% and were removed from analysis. A total of 21 individuals showed evidence of admixture and were consequently excluded. Biological relationship verification revealed a total of 29 sample pairs with cryptic relatedness. For these, the sample with the lower call rate was removed. In addition, further 14 samples with impossible biological sharing or heterogeneity, probably because of contamination, were removed, as well as two individuals who were removed due to gender discrepancies. PC analysis of the remaining individuals for SCES against the HapMap CHB (Han Chinese) reference populations did not show the cohort to be dissimilar in ancestry, and therefore no PCs were used to correct for any underlying population substructure in the analysis performed. Individuals were excluded from the study if they had cataract surgery and missing refraction data. After phenotype and genotype QC, 1,662 individuals were left for the analysis. Logistic regression analyses of astigmatism were performed using 1,662 individuals with age and sex included in the model as covariates.

Strabismus, Amblyopia and Refractive Error Study (STARS)

The Strabismus, Amblyopia and Refractive Error Study in Singaporean Chinese Preschoolers (STARS) Family study is a family-based study nested in a prevalence survey of Singaporean preschool children (n=3,009) conducted from March 2008 to March 2010(Li et al. 2011). The biological parents of STARS probands with myopia (SD ≤ -0.5D) were invited to enroll in the STARS Family study. A total of 1,451 samples from 440 nuclear fmailies were genotyped using Illumina Human610 Quad Beadchips. The 811 parents who were defined as cases/controls for astigmatism and who also had available, high quality GWAS genotypes were used in the current study. The STARS Family study followed the tenets of the Declaration of Helsinki and was approved by Institutional Review Board of the Singapore Eye Research Institute (SERI) and the National Healthcare Group (NHG). Informed written consent was obtained from parents after explanation at clinic. Logistic regression using 811 individuals was used to perform the association analyses of astigmatism with age and sex as covariates.

Singapore Cohort Study of the Risk Factors for Myopia (SCORM)

The Singapore Cohort Study of the Risk Factors for Myopia (SCORM) is a prospective cohort study conducted in two schools in Singapore from 1999-2002. Children aged 7 to 9 years (n = 981) were followed up over a 3-year period. The recruitment and examination procedures have been described

previously (Saw et al. 2002a; Saw et al. 2002b; Saw et al. 2005). The tenets of the Declaration of Helsinki were observed, and approval was granted by the Singapore Eye Research Institute Ethics Committee. Cycloplegic autorefraction and biometry parameter measures were performed annually, according to the same protocol. The astigmatism phenotypes used in this study were based on refractive error measurements from the fourth annual examination. A total of 1116 DNA samples (1037 from buccal swab and 79 from saliva) were genotyped using Illumina HumanHap 550 or 550 Duo Beadarrays (Illumina Inc., San Diego, CA). The Illumina BeadStudio program (Illumina Inc., San Diego, CA) was used for genotyping calls of each marker. Markers were excluded if they were significantly out of Hardy–Weinberg equilibrium in the control dataset ($P<10^{-5}$), had a minor allele frequency <1%, or had missing genotype calls >10% across samples. Samples were excluded if the overall genotype call rate was <98% or deviation in population membership was observed from population structure analysis using EIGENSTRAT programs. After quality control, imputation was performed using IMPUTE software with the reference of Hapmap JPT+CHB populations, build 36, release 22 db126. A total of 917 children with both astigmatism phenotype and high quality genotype data were included in the current analysis. Logistic regression using 917 individuals was used to perform the association analyses of astigmatism with age and sex included as covariates.

All Singapore studies adhere to the Declaration of Helsinki. Ethics approvals have been obtained from the Institutional Review Boards of the Singapore Eye Research Institute, Singapore General hospital, National University of Singapore and National Healthcare Group, Singapore. In all cohorts, participants provided written, informed consent at the recruitment into the studies. For studies involving children (SCORM), written informed consent was obtained from the children's parents.

Supplementary Table S1a. Phenotyping methods – Astigmatism was defined based on refractive error (cylinder power)

	Study name	Measurement of Astigmatism
European adult cohorts	1958 British Birth Cohort	Non-cycloplegic autorefraction
	ALSPAC Mothers	Non-cycloplegic autorefraction
	AREDS1c	subjective refraction
	BATSplusTEST	Humphrey-598 autorefractor
	CROATIA-Korcula	non cycloplegic, Nidek Ark30 hand-held autorefractometer
	CROATIA-Split	non cycloplegic, Nidek Ark30 hand-held autorefractometer
	CROATIA-Vis	non cycloplegic, Nidek Ark30 hand-held autorefractometer
	ERF4	Topcon RM-A2000 autorefractor
	FITSA	Topcon AT (Tokyo, Japan)
	Framingham	subjective refraction
	GUTENBERG	Humphrey-599 automated Refactor
	KORA	Nikon Reinomax
	OGLIASTRA	RK-8100 Topcon autorefractor
	ORCADES	non cyclopegic-Kowa KW 2000 autorefractometer
	ROTTERDAM 1	Topcon RM-A2000 autorefractor
	ROTTERDAM 2	Topcon RM-A2000 autorefractor
	ROTTERDAM 3	Topcon RM-A2000 autorefractor
	TwinsUK	Non-cycloplegic autorefraction
	WESDR adults	subjective refraction
	YFS	NIDEK AR-310AR autorefractor
Asian adult cohorts	BES	Non-cycloplegic autorefraction
	HK-MGS adults	Shin-Nippon SRW-5000
	SCES	Canon RK5 autorefractor
	SIMES	Canon RK-5 Auto Ref-Keratometer
	SINDI	Canon RK-5 Auto Ref-Keratometer
	SP2	Canon RK-5 Auto Ref-Keratometer
	STARS	Canon RK-F1 Autorefractor, Welch Allyn retinoscopy
European youngsters cohorts	ALSPAC children	Non-cycloplegic autorefraction
	BATSplusTEST children	Humphrey-598 autorefractor
	RAINE	Nidek ARK-510A autorefractor
	WESDR children	subjective refraction
Asian youngsters cohort	SCORM	Canon RK5 autorefractor

Supplementary Table S1b. **Genotyping and imputation methods**

	Study name	GWAS chip	Population used for imputation
European adult cohorts	1958 British Birth Cohort	Primarily Illumina 1 million (N=1,000) and then partially overlapping collections of Illumina 610k, Affymetrix 1 million, Affymetrix 500k	CEU (HapMap2)
	ALSPAC Mothers	Illumina 660 W-quad BeadChip	HapMap CEU individuals, build 36, release 22, dbSNP 126
	AREDS1c	Illumina HumanOmni2.5_4v1_B chip array	HapMap2
	BATS & TEST	Illumina HumanHap610 quad	1K Genomes EUR
	CROATIA-Korcula	Illumina 370CNV-Quad	CEU (HapMap2)
	CROATIA-Split	Illumina 370CNV-Quad	CEU (HapMap2)
	CROATIA-Vis	Illumina HumanHap 300v1	CEU (HapMap2)
	ERF4	llumina 6k, Illumina 318K, Illumina 370K and Affymetrix 250K	Hapmap CEU, build 36, release 22
	FITSA	Illumina HumanHap300 (317k)	HapMap2 CEU, release 24
	Framingham	Affy HuGeneFocused_50K; Affy Mapping250k_Nsp; and Affy Mapping250_sty	HapMap2
	GUTENBERG	Affymetrix Genome-Wide Human SNP6.0 Array	HapMap2
	KORA	HumanOmni2.5-4v1_B chip array	HapMap2
	OGLIASTRA	500k Affymetrix array chip	CEU build 36 release 22
	ORCADES	part Illumina HumanHap 300v2 and part 370CNV-Quad	HapMap2 CEU
	ROTTERDAM 1	Illumina Infinium II HumanHap550 chip v3.0 array	Hapmap CEU, build 36, release 22
	ROTTERDAM 2	HumanHap550 Duo Arrays + Human610-Quad Arrays Illumina	Hapmap CEU, build 36, release 22
	ROTTERDAM 3	Human 610 Quad Arrays Illumina	Hapmap CEU, build 36, release 22
	TwinsUK	Illumina 610k, Illumina 317K	CEU (HapMap2)
	WESDR adults	Illumina Human Omni1-Quad	HapMap 2 r22 CEU+CHB+JPT+YRI (IMPUTE automatically picks the best custom set of reference panel for each individual)
	YFS	Illumina HumanHap670k BeadChip	Hapmap2 CEF (release 22, build 36, dbSNP 126

Supplementary Table S1b continued…

**Asian adult cohorts**	BES	Illumina HumanHap550 BeadChip	HapMap CEU individuals, build 36, release 22, dbSNP 126
	HK-MGS adults	Illumina human610-quad	HapMap Asian panel
	SCES	HumanHap 610	Hapmap JPT+CHB, build 36, release 22 db126
	SIMES	HumanHap 610	Hapmap CEU, JPT+CHB and YRI, build 36, release 22 db126
	SINDI	HumanHap 610	Hapmap CEU, JPT+CHB and YRI, build 36, release 22 db127
	SP2	HumanHap 610,1million	Hapmap JPT+CHB, build 36, release 22 db126
	STARS	HumanHap 610	Hapmap JPT+CHB, build 36, release 22 db126
**European youngsters cohorts**	ALSPAC children	Illumina 660 W-quad BeadChip	HapMap CEU individuals, build 36, release 22, dbSNP 126
	BATS & TEST children	Illumina HumanHap610 quad	1K Genomes EUR
	RAINE	Illumina 660W Quad	HapMap2
	WESDR children	Illumina Human Omni1-Quad	HapMap2 r22 CEU+CHB+JPT+YRI
**Asian youngsters cohort**	SCORM	HumanHap 550,550Duo	Hapmap JPT+CHB, build 36, release 22 db126

Supplementary Table S2. Study specific lambda estimates

	Study Name	lambda
European adult cohorts	1958 British Birth Cohort	1.006
	ALSPAC Mothers	1.004
	AREDS	1.000
	BATS & TEST adults	1.011
	CROATIA-Korcula	1.000
	CROATIA-Split	0.995
	CROATIA-Vis	0.997
	ERF4	1.006
	FITSA	1.094
	Framingham	0.982
	GUTENBERG	0.993
	KORA	0.982
	OGLIASTRA	0.986
	ORCADES	0.998
	ROTTERDAM 1	0.998
	ROTTERDAM 2	1.001
	ROTTERDAM 3	0.997
	TwinsUK	0.996
	WESDR adults	0.999
	YFS	0.998
Asian adult cohorts	BES	0.995
	HK-MGS adults	0.997
	SCES	0.999
	SIMES	0.998
	SINDI	0.998
	SP2	1.003
	STARS	0.997
European yourgesters cohorts	ALSPAC children	1.001
	BATS & TEST children	1.013
	RAINE	1.002
	WESDR children	1.010
Asian youngsters cohort	SCORM	0.998

Additional authors and affiliations

Nobuhisa Mizuki [1], Nagahisa Yoshimura [2], Kenji Yamashiro [2], Margaret Deangelis [3], Xiangtian Zhou [4], Cécile Delcourt [5,6].

1. Department of Ophthalmology, Yokohama City University School of Medicine, Yokohama, Kanagawa, Japan.
2. Department of Ophthalmology and Visual Sciences, Kyoto University Graduate School of Medicine, Kyoto, Japan.
3. Department of Ophthalmology and Visual Sciences, University of Utah, Moran Eye Center, Salt Lake City, USA.
4. School of Optometry and Ophthalmology and Eye Hospital, Wenzhou Medical College, Wenzhou, Zhejiang, China
5. Université de Bordeaux, Bordeaux, France.
6. INSERM (Institut National de la Santé Et de la Recherche Médicale), ISPED (Institut de Santé Publique d'Épidémiologie et de Développement), Centre INSERM U897-Epidemiologie-Biostatistique, Bordeaux, France.

Supplementary acknowledgements

We would like to acknowledge the following agencies and persons:

1958 British Birth Cohort phenotyping was funded by the Medical Research Council's Health of the Public grant (PIs Power and Strachan); the genetic studies by the Wellcome Trust (083478 to J.S.R.); some of the analysis by the National Institute for Health Research as Specialist Biomedical Research Centres in Paediatrics and Ophthalmology, partnering respectively with Great Ormond Street and Moorfields Hospitals; with additional personal funding (P.M.C) by the Ulverscroft Vision Research Group.

Core support for **ALSPAC** was provided by the UK Medical Research Council (4882); the Wellcome Trust (076467); the University of Bristol; and for this research specifically by the National Eye Research Centre, Bristol (SCIAD053); C.W. is supported by an NIHR Fellowship. ALSPAC thanks all the families who took part in this study, the midwives for their help in recruiting them, and the whole ALSPAC team, which includes interviewers, computer and laboratory technicians, clerical workers, research scientists, volunteers, managers, receptionists and nurses.

Support for recruitment of **ANZRAG** was provided by the Royal Australian and New Zealand College of Ophthalmology (RANZCO) Eye Foundation. Genotyping was funded by the National Health and Medical Research Council of Australia (#535074). The authors acknowledge the support of Ms Bronwyn Usher-Ridge and Ms Emmanuelle Souzeau in patient recruitment and data collection, Professor Matthew A.Brown and Dr Patrick Danoy for genotyping and Mr Rhys Fogarty for data analysis.

AREDS1a1b and **FECD** were supported by the National Eye Institute (grants R01EY16482, R21EY015145, and P30EY11373) and by Research to Prevent Blindness and the Ohio Lions Eye Research Foundation. The investigators gratefully acknowledge the role of the clinical co-ordinators and investigators who collected data on FECD cases and controls. Individual investigators and sites are listed in the first publication of the FECD study (Louttit et al. 2012). Data for the AREDS1a and 1b studies was downloaded from dbGaP for analysis under a National Eye Institute data use agreement.

AREDS1c was supported by contracts from National Eye Institute/National Institutes of Health, Bethesda, MD, with additional support from Bausch & Lomb Inc, Rochester, NY. The genotyping costs were supported by the National Eye Institute (R01EY020483 to D.S.) and some of the analyses were supported by the Intramural Research Program of the National Human Genome Research Institute, National Institutes of Health, USA. AREDS acknowledges Frederick Ferris, National

Eye Institute, National Institutes of Health, Bethesda, MD; and the Center for Inherited Disease Research, Baltimore, MD where SNP genotyping was carried out. The investigators gratefully acknowledge the advice and guidance of Hemin Chin of the National Eye Institute.

BMES was supported by the Australian National Health & Medical Research Council (NH&MRC), Canberra Australia (974159, 211069, 457349, 512423, 475604, 529912); the Centre for Clinical Research Excellence in Translational Clinical Research in Eye Diseases; NH&MRC research fellowships (358702, 632909 to J.J.W, 1028444 to P.N.B.); and the Wellcome Trust, UK as part of Wellcome Trust Case Control Consortium 2 (A. Viswanathan, P. McGuffin, P. Mitchell, F. Topouzis, P. Foster) for genotyping costs of the entire BMES population (085475B08Z, 08547508Z, 076113). The Centre for Eye Research Australia receives Operational Infrastructure Support from the Victorian government. BMES acknowledges Elena Rochtchina from the Centre for Vision Research, Department of Ophthalmology and Westmead Millennium Institute University of Sydney (NSW Australia); John Attia, Rodney Scott, Elizabeth G. Holliday from the University of Newcastle (Newcastle, NSW Australia); Jing Xie, and Andrea J. Richardson from the Centre for Eye Research Australia, University of Melbourne; Michael T. Inouye, Medical Systems Biology, Department of Pathology & Department of Microbiology & Immunology, University of Melbourne (Victoria, Australia); Ananth Viswanathan, Moorfields Eye Hospital (London, UK); Paul J. Foster, NIHR Biomedical Research Centre for Ophthalmology, UCL Institute of Ophthalmology & Moorfields Eye Hospital (London); Peter McGuffin, MRC Social Genetic and Developmental Psychiatry Research Centre, Institute of Psychiatry, King's College (London, United Kingdom); Fotis Topouzis, Department of Ophthalmology, School of Medicine, Aristotle University of Thessaloniki, AHEPA Hospital (Thessaloniki, Greece); Xueling Sim, National University of Singapore; members of the Wellcome Trust Case Control Consortium 2 (see **Supplementary Information**).

The **CROATIA** studies were funded by grants from the Medical Research Council (UK) and from the Republic of Croatia Ministry of Science, Education and Sports (10810803150302); and the CROATIA-Korcula genotyping was funded by the European Union framework program 6 project EUROSPAN (LSHGCT2006018947). The CROATIA studies acknowledges Dr. Goran Bencic, Biljana Andrijević Derk, Valentina Lacmanović Lončar, Krešimir Mandić, Antonija Mandić, Ivan Škegro, Jasna Pavičić Astaloš, Ivana Merc, Miljenka Martinović, Petra Kralj, Tamara Knežević and Katja Barać-Juretić as well as the recruitment team from the Croatian Centre for Global Health, University of Split and the Institute of Anthropological Research in Zagreb for the ophthalmological data collection; Peter Lichner and the Helmholtz Zentrum Munchen (Munich, Germany), AROS Applied Biotechnology, Aarhus, Denmark and the Wellcome Trust Clinical facility (Edinburgh, United Kingdom) for the SNP

genotyping all studies; Jennifer Huffman, Susan Campbell and Pau Navarro for genetic data preparation.

EGCUT received financing by FP7 grants (201413, 245536); Estonian Government (SF0180142s08); and the European Union through the European Regional Development Fund, in the frame of Centre of Excellence in Genomics and Estonian Research Infrastructure's Roadmap; EFSD grant; and the University of Tartu (SP1GVARENG). EGCUT acknowledges Mr. T. Esko, Mr. V. Soo, Ms. M-L. Tammesoo.

FITSA was supported by ENGAGE (FP7-HEALTH-F4-2007, 201413); European Union through the GENOMEUTWIN project (QLG2-CT-2002-01254); the Academy of Finland Center of Excellence in Complex Disease Genetics (213506, 129680); the Academy of Finland Ageing Programme; and the Finnish Ministry of Culture and Education and University of Jyväskylä. For FITSA the contributions of Emmi Tikkanen, Samuli Ripatti, Markku Kauppinen, Taina Rantanen and Jaakko Kaprio are acknowledged.

Framingham Eye Study is a sub-study of the Framingham Heart Study, which is conducted and supported by the National Heart, Lung, and Blood Institute (NHLBI) in collaboration with Boston University (Contract No. N01-HC-25195). These data were obtained from the NIH repository dbGaP (accession numbers phs000007/HMB-IRB-MDS and phs000007/HMB-IRB-NPU-MDS). This manuscript was not prepared in collaboration with investigators of the Framingham Heart Study and does not necessarily reflect the opinions or views of the Framingham Heart Study, Boston University, or NHLBI. Funding for SHARe Affymetrix genotyping was provided by NHLBI Contract N02-HL-64278. SHARe Illumina genotyping was provided under an agreement between Illumina and Boston University. Funding for the collection of FES phenotype data was supported by the National Eye Institute (ZIAEY000403; N01EY22112, N01EY92109). The current analyses were supported by intramural funds of the National Human Genome Research Institute, NIH, USA (to JEBW, QL, CLS, and RW).

The **Myopia Genomics Study** was supported by grants from the Hong Kong Polytechnic University (J-BB7P, G-U730, 87TP and 87U7) and the Research Grant Council of Hong Kong (B-Q33T).

The Gutenberg Health Study was funded through the government of Rheinland-Pfalz ("Stiftung Rheinland Pfalz für Innovation" (AZ961386261733); the research programs "Wissen schafft Zukunft" and "Schwerpunkt Vaskuläre Prävention" of the Johannes Gutenberg-University of Mainz; Boehringer Ingelheim; PHILIPS Medical Systems; National Genome Network ''NGFNplus'' by the Federal Ministry of Education and Research, Germany (A301GS0833). GHS acknowlegdes Dagmar

Laubert-Reh for data analysis.

KORA was financed by the Helmholtz Center Munich, German Research Center for Environmental Health; the German Federal Ministry of Education and Research; the State of Bavaria; the German National Genome Research Network (NGFN-2 and NGFNPlus) (01GS0823); Munich Center of Health Sciences as part of LMUinnovativ; the genotyping was carried out by the Center for Inherited Disease Research, Baltimore, MD, and was supported by the National Eye Institute (R01 EY020483 to D.S.). Some of the analyses were supported by the Intramural Research Program of the National Human Genome Research Insitute, NIH, USA.

OGP Talana was supported by grants from the Italian Ministry of Education, University and Research (5571DSPAR2002, 718Ric2005). OGP Talana thanks the Ogliastra population and the municipal administrators for their collaboration to the project and for economic and logistic support.

ORCADES was supported by the Chief Scientist Office of the Scottish Government, the Royal Society, the Medical Research Council Human Genetics Unit and the European Union framework program 6 EUROSPAN project (LSHGCT2006018947). ORCADES acknowledges the invaluable contributions of Lorraine Anderson and the research nurses in Orkney, in particular Margaret Pratt who performed the eye measurements,as well as the administrative team in Edinburgh University; and the Wellcome Trust Clinical facility (Edinburgh, United Kingdom) for DNA extraction; and Peter Lichner and the Helmholtz Zentrum Munchen (Munich, Germany) for genotyping; and Mirna Kirin for the genetic data imputation.

The Rotterdam Study and **ERF** were supported by the Netherlands Organisation of Scientific Research (NWO) (Vidi 91796357); Erasmus Medical Center and Erasmus University, Rotterdam, The Netherlands; Netherlands Organization for Health Research and Development (ZonMw); UitZicht; the Research Institute for Diseases in the Elderly; the Ministry of Education, Culture and Science; the Ministry for Health, Welfare and Sports; the European Commission (DG XII); the Municipality of Rotterdam; the Netherlands Genomics Initiative/NWO; Center for Medical Systems Biology of NGI; Lijf en Leven; M.D. Fonds; Henkes Stichting; Stichting Nederlands Oogheelkundig Onderzoek; Swart van Essen; Bevordering van Volkskracht; Blindenhulp; Landelijke Stichting voor Blinden en Slechtzienden; Rotterdamse Vereniging voor Blindenbelangen; OOG; Algemene Nederlandse Vereniging ter Voorkoming van Blindheid; the Rotterdam Eye Hospital Research Foundation; Topcon Europe; Ada Hooghart, Corina Brussee, Riet Bernaerts-Biskop, Patricia van Hilten, Pascal Arp, Jeanette Vergeer, Marijn Verkerk; Sander Bervoets; Daan Loth and Maarten Kooijman.

TEST and **BATS** (Australian Twins) were supported by an Australian National Health and Medical Research Council (NHMRC) Enabling Grant (2004-2009, 350415, 2005-2007); Clifford Craig Medical Research Trust; Ophthalmic Research Institute of Australia; American Health Assistance Foundation; Peggy and Leslie Cranbourne Foundation; Foundation for Children; Jack Brockhoff Foundation; National Institutes of Health/National Eye Institute (RO1EY01824601 (2007-2010)); Pfizer Australia Senior Research Fellowship (to D.A.M.); and Australian NHMRC Career Development Award (to S.M.). Genotyping was funded by an NHMRC Medical Genomics Grant; US National Institutes of Health/National Eye Institute (1RO1EY018246), Australian sample imputation analyses were carried out on the Genetic Cluster Computer which is financially supported by the Netherlands Scientific Organization (NWO48005003). Australian Twins thanks Nicholas Martin, Scott Gordon, Dale Nyholt, Sarah Medland, Brian McEvoy, Margaret Wright, Anjali Henders, Megan Campbell for ascertaining and processing genotyping data; Jane MacKinnon, Shayne Brown, Lisa Kearns, Jonathan Ruddle, Paul Sanfilippo, Sandra Staffieri, Olivia Bigault, Colleen Wilkinson, Jamie Craig, Yaling Ma, Julie Barbour for assisting with clinical examinations; and Dr Camilla Day and staff at the Center for Inherited Disease Research.

TwinsUK received funding from the Wellcome Trust; the European Union MyEuropia Marie Curie Research Training Network; Guide Dogs for the Blind Association; the European Community's FP7 (HEALTHF22008201865GEFOS); ENGAGE (HEALTHF42007201413); the FP-5 GenomEUtwin Project (QLG2CT200201254); US National Institutes of Health/National Eye Institute (1RO1EY018246); NIH Center for Inherited Disease Research; the National Institute for Health Research comprehensive Biomedical Research Centre award to Guy's and St. Thomas' National Health Service Foundation Trust partnering with King's College London. P.G.H. is the recipient of a Fight for Sight ECI award. We acknowledge the contribution of Drs Toby Andrew, Margarida Lopes, Samantha Fahy and Diana Kozareva.

WESDR was supported by NEI (grants R01EY03083 and EY016379) and a Research to Prevent Blindness Senior Scientific Investigator Award.

Young Finns Study was financially supported by the Academy of Finland (134309 (Eye), 126925, 121584, 124282, 129378 (Salve), 117787 (Gendi), and 41071 (Skidi)); the Social Insurance Institution of Finland, Kuopio, Tampere; Turku University Hospital Medical Funds (grant 9M048 and 9N035 to T.L.); Juho Vainio Foundation; Paavo Nurmi Foundation; Finnish Foundation of Cardiovascular Research and Finnish Cultural Foundation; Tampere Tuberculosis Foundation; and

Emil Aaltonen Foundation (to T.L.). The expert technical assistance in the statistical analyses by Irina Lisinen and Ville Aalto are gratefully acknowledged.

Beijing Eye Study was supported by National Natural Science Foundation of China (grant 81170890).

The core management of the **Raine** Study is funded by The University of Western Australia (UWA), The Telethon Institute for Child Health Research, Raine Medical Research Foundation, UWA Faculty of Medicine, Dentistry and Health Sciences, Women's and Infant's Research Foundation and Curtin University. Genotyping was funded by NHMRC project grant 572613. Support for the Raine Eye Health Study was provided by NHMRC Grant 1021105, Lions Eye Institute, the Australian Foundation for the Prevention of Blindness and Alcon Research Institute. The Raine Eye Health Study authors thank the Raine eye health study participants and their families. They also thank the Raine Study management, Craig Pennell, Jenny Mountain and the team at TICHR and LEI for cohort co-ordination and data collection, particularly: Seyhan Yazar, Hannah Forward,Charlotte McKnight, Alex Tan, Alla Soloshenko, Sandra Oates, and Diane Wood.

The Singapore studies (**SCORM, STARS, SP2, SIMES, SINDI, SCES**) were supported by the National Medical Research Council, Singapore (NMRC 0796/2003, NMRC 1176/2008, STaR/0003/2008; CG/SERI/2010), Biomedical Research Council, Singapore (06/1/21/19/466, 09/1/35/19/616 and 08/1/35/19/550). The Singapore Tissue Network and the Genome Institute of Singapore, Agency for Science, Technology and Research, Singapore provided services.

The Strabismus, Amblyopia, and Refractive Error Study of Preschool Children (**STARS**) was supported by a NMRC grant (1176/2008). Ching-Yu Cheng is supported by an award from NMRC (CSA/033/2012). E-Shyong Tai is also supported by an award from NMRC (CSA/008/2009). The Singapore Tissue Network and the Genome Institute of Singapore, Agency for Science, Technology and Research, Singapore provided services for tissue archival and genotyping, respectively.

We would like to acknowledge the CREAM consortium members:

The Consortium for Refractive Error and Myopia (CREAM)

**Study**	**Members**
1958 British Birth Cohort	Christopher J. Hammond[1], Pirro G. Hysi[1], Jugnoo S. Rahi[2-4]
Aichi cohort	Nagahisa Yoshimura[5], Kenji Yamashiro[5], Masahiro Miyake[5]
ALIENOR	Cécile Delcourt[6,7], Jean-François Korobelnik[6,7]
ALSPAC	Cathy Williams[8], Jeremy A. Guggenheim[9], George McMahon[10], John P. Kemp[10], Beate St Pourcain[10], David M. Evans[10,11], Nicholas J. Timpson[10], George Davey Smith[10]
ANZRAG	Jamie E. Craig[12], Kathryn P. Burdon[12], Rhys D. Fogarty[12]
AREDS1ab	Sudha K. Iyengar[13-15], Robert P. Igo J[13], Emily Chew[16], Sarayut Janmahasatian[13]
AREDS1c	Dwight Stambolian[17], Joan E. Bailey Wilson[18]
BATS	Nicholas G. Martin[19], Yi Lu[20], Stuart MacGregor[20], David Mackey[21,22], Alex Hewitt[21,22]
Beijing Eye Study	Jost B. Jonas[23,24], Ya Xing Wang[24], Liang Xu[24], Seang Mei Saw[25-28]
Blue Mountain Eye Study	Paul N. Baird[21], Maria Schache[21], Paul Mitchell[29], Jie Jin Wang[21,29], Jing Xie[21]
CIEMS	Vinay Nangia[30], Songhomitra Panda-Jonas[30]
CROATIA-Korčula	Caroline Hayward[31], Alan F. Wright[31], Veronique Vitart[31]
CROATIA-Split	Ozren Polasek[32], Harry Campbell[33], Veronique Vitart[31]
CROATIA-Vis	Igor Rudan[33], Zoran Vatavuk[34], Veronique Vitart[31]
CWRU FECD Fuchs Dystrophy	Sudha K. Iyengar[13-15], Robert P. Igo Jr[13], Jeremy R. Fondran[13], Jonathan H. Lass[13,14]
DCCT	Andrew D. Paterson[35], S. Mohsen Hosseini[35]

Duke Myopia Study	Terri L. Young[36], Sheng Feng[36]
EPIC-Norfolk	Robert N. Luben[37], Jing Hua Zhao[38], Anthony P. Khawaja[37], Paul J. Foster[39,40], Kay-Tee Khaw[37], Nick J. Wareham[41]
Estonian Genome Center	Andres Metspalu[42], Toomas Haller[42], Evelin Mihailov[42]
FITSA	Taina Rantanen[43,44], Jaakko Kaprio[45-47],Olavi Pärssinen[43,44,48], Juho Wedenoja[49,50]
Framingham Eye Studies	Joan E. Bailey Wilson[18], Robert Wojciechowski[18,51], Claire L. Simpson[18]
GEMT	Paul N. Baird[21], Maria Schache[21]
Guangzhou High Myopia Registry	Mingguang He[52], Xiaobo Guo[52]
Guangzhou Twin Eye Study	Mingguang He[52], Xiaobo Guo[52]
Gutenberg Health Study	Norbert Pfeiffer[53], René Höhn[53]
Hong Kong cohort study	Chi Pui Pang[54], Li Jia Chen[55], Pancy O. Tam[54], Vishal Jhanji[54,55], Alvin L. Young[54,55]
KORA	Thomas Meitinger[56], Konrad Oexle[56], Janina S. Ried[57], Angela Döring[58,59] and Christian Gieger[57]
Kyoto high myopia	Nagahisa Yoshimura[5], Kenji Yamashiro[5], Masahiro Miyake[5]
LIKI	Olavi Pärssinen[43,44,48]
MESA	Leslie J. Raffel[60], Mary-Frances Cotch[61], Barbara E. Klein[62], Ronald Klein[62], Xiaohui Li[63]
Myopia Genomics Study (Hong Kong HTI)	Shea Ping Yip[64], Maurice K.H. Yap[64]
Nagahama Study	Nagahisa Yoshimura[5], Kenji Yamashiro[5], Masahiro Miyake[5]
Ogliastra Genetic Park Study	Mario Pirastu[65], Federico Murgia[65], Laura Portas[65], Ginevra Biino[66], Maurizio Fossarello[65]
ORCADES	James F. Wilson[33], Brian Fleck[67], Veronique Vitart[31]
Penn Family Studies	Dwight Stambolian[17], Joan E. Bailey Wilson[18]
RAINE	Alex W. Hewitt[21,22], Seyhan Yazar[21,22], David Mackey[21,22], Stuart MacGregor[20], Puya Gharahkhani[20]
Rotterdam Studies (Rotterdam Study I-III, Erasmus Rucphen Family Study,	Virginie J.M. Verhoeven[68,69], Caroline C. Klaver[68,69], Cornelia M. van Duijn[69], Jan Willem L. Tideman[68,69]

Generation R, MYST)	
Singapore Studies (SCES, SCORM, SIMES, SINDI, SP2, STARS)	Seang-Mei Saw[25-28], Qiao Fan[25], Peng Chen[25], Jiemin Liao[27], Amutha Barathi Veluchamy[26-28], M. Kamran Ikram[25-27], E-Shyong Tai[25,28,70], Tin Aung[26,27], Chiea-Chuen Khor[25,27,71,72], Yik-Ying Teo[25,73], Ching-Yu Cheng[25-27], Tien-Yin Wong[25-27]
TEST	David A. Mackey[21,22], Stuart MacGregor[20], Alex Hewitt[21,22]
TwinsUK & 1958 British Birth Cohort	Christopher J. Hammond[1], Pirro G. Hysi[1], Jugnoo S. Rahi[2-4]
Utah Timorese	Margaret M. Deangelis[74], Margaux Morrison[75], Lindsay Farrer[76]
Wenzhou	Xiangtian Zhou[77], Wei Chen[77]
WESDR	Andrew D. Paterson[35], S. Mohsen Hosseini[35], Barbara E. Klein[62], Ronald Klein[62]
Yokohama Study	Nobuhisa Mizuki[78], Akira Meguro[78]
Young Finns Study	Terho Lehtimäki[79], Kari Matti Mäkelä[79], Olli Raitakari[80,81], Mika Kähönen[82], Ilkka Seppala[79]

Affiliations

1. Department of Twin Research and Genetic Epidemiology, King's College London School of Medicine, London, UK.
2. Medical Research Council Centre of Epidemiology for Child Health, Institute of Child Health, University College London, London, UK.
3. Institute of Ophthalmology, Moorfields Eye Hospital, London, UK.
4. Ulverscroft Vision Research Group, University College London, London, UK.
5. Department of Ophthalmology and Visual Sciences, Kyoto University Graduate School of Medicine, Kyoto, Japan.
6. Université de Bordeaux, Bordeaux, France.
7. INSERM (Institut National de la Santé Et de la Recherche Médicale), ISPED (Institut de Santé Publique d'Épidémiologie et de Développement), Centre INSERM U897-Epidemiologie-Biostatistique, Bordeaux, France.
8. School of Social and Community Medicine, University of Bristol, Bristol, UK.
9. Centre for Myopia Research, School of Optometry, The Hong Kong Polytechnic University, Hong Kong, Hong Kong.
10. MRC Integrative Epidemiology Unit (IEU), the University of Bristol, Bristol, UK. .
11. University of Queensland Diamantina Institute, Translational Research Institute, Brisbane, Queensland, Australia.
12. Department of Ophthalmology, Flinders University, Adelaide, Australia.
13. Department of Epidemiology and Biostatistics, Case Western Reserve University, Cleveland, Ohio, USA.
14. Department of Ophthalmology and Visual Sciences, Case Western Reserve University and University Hospitals Eye Institute, Cleveland, Ohio, USA.
15. Department of Genetics, Case Western Reserve University, Cleveland, Ohio, USA.
16. National Eye Institute, National Institutes of Health, Bethesda, Maryland, USA.

17. Department of Ophthalmology, University of Pennsylvania, Philadelphia, Pennsylvania, USA.
18. Inherited Disease Research Branch, National Human Genome Research Institute, National Institutes of Health, Baltimore, Maryland, USA.
19. Genetic Epidemiology Laboratory, QIMR Berghofer Medical Research Institute, Herston, Brisbane, Queensland, Australia.
20. Statistical Genetics Laboratory, QIMR Berghofer Medical Research Institute, Herston, Brisbane, Queensland, Australia.
21. Centre for Eye Research Australia (CERA), University of Melbourne, Royal Victorian Eye and Ear Hospital, Melbourne, Victoria, Australia.
22. Centre for Ophthalmology and Visual Science, Lions Eye Institute, University of Western Australia, Perth, Australia.
23. Department of Ophthalmology, Medical Faculty Mannheim, Ruprecht-Karls-University Heidelberg, Mannheim, Germany.
24. Beijing Institute of Ophthalmology, Beijing Tongren Hospital, Capital Medical University, Beijing, China.
25. Saw Swee Hock School of Public Health, National University Health Systems, National University of Singapore, Singapore, Singapore.
26. Singapore Eye Research Institute, Singapore National Eye Centre, Singapore, Singapore.
27. Department of Ophthalmology, National University Health Systems, National University of Singapore, Singapore.
28. DUKE-National University of Singapore Graduate Medical School, Singapore, Singapore.
29. Department of Ophthalmology, Centre for Vision Research, Westmead Millennium Institute, University of Sydney, Sydney, Australia.
30. Suraj Eye Institute, Nagpur, Maharashtra, India.
31. Medical Research Council Human Genetics Unit, Institute of Genetics and Molecular Medicine, University of Edinburgh, Edinburgh, UK.
32. Faculty of Medicine, University of Split, Split, Croatia.
33. Centre for Population Health Sciences, University of Edinburgh, Edinburgh, UK.
34. Department of Ophthalmology, Sisters of Mercy University Hospital, Zagreb, Croatia.
35. Program in Genetics and Genome Biology, Hospital for Sick Children and University of Toronto, Toronto, Ontario, Canada.
36. Department of Pediatric Ophthalmology, Duke Eye Center For Human Genetics, Durham, North Carolina, USA.
37. Department of Public Health and Primary Care, Institute of Public Health, University of Cambridge School of Clinical Medicine, Cambridge, UK. .
38. MRC Epidemiology Unit, Institute of Metabolic Sciences, University of Cambridge, Cambridge, UK.
39. Division of Genetics and Epidemiology, UCL Institute of Ophthalmology, London, UK.
40. NIHR Biomedical Research Centre, Moorfields Eye Hospital NHS Foundation Trust and UCL Institute of Ophthalmology, London, UK.
41. MRC Epidemiology Unit, Institute of Metabolic Science, Addenbrooke's Hospital, Cambridge, UK.
42. Estonian Genome Center, University of Tartu, Tartu, Estonia.
43. Gerontology Research Center and Department of Health Sciences, University of Jyväskylä, Jyväskylä, Finland.
44. Gerontology Research Center, University of Jyväskylä, Jyväskylä, Finland.
45. Department of Public Health, University of Helsinki, Helsinki, Finland.
46. Institute for Molecular Medicine, University of Helsinki, Helsinki, Finland.
47. Department of Mental Health and Alcohol Abuse Services, National Institute for Health and Welfare, Helsinki, Finland.
48. Department of Ophthalmology, Central Hospital of Central Finland, Jyväskylä, Finland.
49. Department of Public Health, Hjelt Institute, University of Helsinki, Finland.

50. Department of Ophthalmology, Helsinki University Central Hospital, Helsinki, Finland.
51. Department of Epidemiology, Johns Hopkins Bloomberg School of Public Health, Baltimore, Maryland, USA.
52. State Key Laboratory of Ophthalmology, Zhongshan Ophthalmic Center, Sun Yat-sen University, Guangzhou, China.
53. Department of Ophthalmology, University Medical Center Mainz, Mainz, Germany.
54. Department of Ophthalmology and Visual Sciences, The Chinese University of Hong Kong Hong Kong Eye Hospital, Kowloon, Hong Kong.
55. Department of Ophthalmology and Visual Sciences, The Chinese University of Hong Kong, Prince of Wales Hospital, Shatin, Hong Kong. .
56. Institute of Human Genetics, Technical University Munich, Munich, Germany.
57. Institute of Genetic Epidemiology, Helmholtz Zentrum München—German Research Center for Environmental Health, Neuherberg, Germany.
58. Institute of Epidemiology I, Helmholtz Zentrum München, German Research Center for Environmental Health, Neuherberg, Germany.
59. Institute of Epidemiology II, Helmholtz Zentrum München, German Research Center for Environmental Health, Neuherberg, Germany.
60. Medical Genetics Institute, Cedars-Sinai Medical Center, Los Angeles, CA, USA.
61. Division of Epidemiology and Clinical Applications, National Eye Institute, Bethesda, Maryland, USA.
62. Department of Ophthalmology and Visual Sciences, University of Wisconsin School of Medicine and Public Health, Madison, WI, USA.
63. Institute for Translational Genomics and Population Sciences, Los Angeles Biomedical Research Institute, Harbor-UCLA Medical Center, United States.
64. Department of Health Technology and Informatics, The Hong Kong Polytechnic University, Hong Kong, Hong Kong. .
65. Institute of Population Genetics, National Research Council, Sassari, Italy.
66. Molecular Genetics, National Research Council, Pavia, Italy.
67. Princess Alexandra Eye Pavilion, Edinburgh, UK.
68. Department of Ophthalmology, Erasmus Medical Center, Rotterdam, The Netherlands.
69. Department of Epidemiology, Erasmus Medical Center, Rotterdam, The Netherlands.
70. Department of Medicine, National University of Singapore, Singapore, Singapore.
71. Department of Pediatrics, National University of Singapore, Singapore, Singapore.
72. Division of Human Genetics, Genome Institute of Singapore, Singapore, Singapore.
73. Department of Statistics and Applied Probability, National University of Singapore, Singapore, Singapore.
74. Department of Ophthalmology and Visual Sciences, University of Utah, Moran Eye Center, Salt Lake City, USA.
75. Department of Ophthalmology and Visual Sciences, John Moran Eye Center, University of Utah, 60 North Mario Capecchi Dr., Salt Lake City, Utah, 84132, USA.
76. Departments of Medicine (Biomedical Genetics), Ophthalmology, Neurology, Epidemiology, and Biostatistics, Boston University Schools of Medicine and Public Health, Boston, USA.
77. School of Optometry and Ophthalmology and Eye Hospital, Wenzhou Medical College, Wenzhou, Zhejiang, China
78. Department of Ophthalmology, Yokohama City University School of Medicine, Yokohama, Kanagawa, Japan.
79. Department of Clinical Chemistry, Fimlab laboratories and School of Medicine, University of Tampere, Tampere, Finland.
80. Research Centre of Applied and Preventive Medicine, University of Turku, Turku, Finland.
81. Department of Clinical Physiology and Nuclear Medicine, Turku University Hospital, Turku, Finland.

82. Department of Clinical Physiology, Tampere University Hospital and School of Medicine, University of Tampere, Tampere, Finland.

References

Age-Related Eye Disease Study Research Group (1999) The Age-Related Eye Disease Study (AREDS): design implications. AREDS report no. 1. Control Clin Trials 20: 573-600

Age-Related Eye Disease Study Research Group (2001a) A randomized, placebo-controlled, clinical trial of high-dose supplementation with vitamins C and E and beta carotene for age-related cataract and vision loss: AREDS report no. 9. Arch Ophthalmol 119: 1439-52

Age-Related Eye Disease Study Research Group (2001b) A randomized, placebo-controlled, clinical trial of high-dose supplementation with vitamins C and E, beta carotene, and zinc for age-related macular degeneration and vision loss: AREDS report no. 8. Arch Ophthalmol 119: 1417-36

Aulchenko YS, Heutink P, Mackay I, Bertoli-Avella AM, Pullen J, Vaessen N, Rademaker TA, Sandkuijl LA, Cardon L, Oostra B, van Duijn CM (2004) Linkage disequilibrium in young genetically isolated Dutch population. Eur J Hum Genet 12: 527-34

Biino G, Palmas MA, Corona C, Prodi D, Fanciulli M, Sulis R, Serra A, Fossarello M, Pirastu M (2005) Ocular refraction: heritability and genome-wide search for eye morphometry traits in an isolated Sardinian population. Hum Genet 116: 152-9

Booij JC, Bakker A, Kulumbetova J, Moutaoukil Y, Smeets B, Verheij J, Kroes HY, Klaver CC, van Schooneveld M, Bergen AA, Florijn RJ (2011) Simultaneous mutation detection in 90 retinal disease genes in multiple patients using a custom-designed 300-kb retinal resequencing chip. Ophthalmology 118: 160-167 e1-3

Boyd A, Golding J, Macleod J, Lawlor DA, Fraser A, Henderson J, Molloy L, Ness A, Ring S, Davey Smith G (2013) Cohort Profile: the 'children of the 90s'--the index offspring of the Avon Longitudinal Study of Parents and Children. International Journal of Epidemiology 42: 111-27

Clemons TE, Chew EY, Bressler SB, McBee W, Age-Related Eye Disease Study Research G (2003) National Eye Institute Visual Function Questionnaire in the Age-Related Eye Disease Study (AREDS): AREDS Report No. 10. Arch Ophthalmol 121: 211-7

Cornes BK, Khor CC, Nongpiur ME, Xu L, Tay WT, Zheng Y, Lavanya R, Li Y, Wu R, Sim X, Wang YX, Chen P, Teo YY, Chia KS, Seielstad M, Liu J, Hibberd ML, Cheng CY, Saw SM, Tai ES, Jonas JB, Vithana EN, Wong TY, Aung T (2012) Identification of four novel variants that influence central corneal thickness in multi-ethnic Asian populations. Hum Mol Genet 21: 437-45

Foong AW, Saw SM, Loo JL, Shen S, Loon SC, Rosman M, Aung T, Tan DT, Tai ES, Wong TY (2007) Rationale and methodology for a population-based study of eye diseases in Malay people: The Singapore Malay eye study (SiMES). Ophthalmic Epidemiol 14: 25-35

Fraser A, Macdonald-Wallis C, Tilling K, Boyd A, Golding J, Davey Smith G, Henderson J, Macleod J, Molloy L, Ness A, Ring S, Nelson SM, Lawlor DA (2013) Cohort Profile: the Avon Longitudinal Study of Parents and Children: ALSPAC mothers cohort. International Journal of Epidemiology 42: 97-110

Framingham Offspring Eye Study Group (1996) Familial aggregation and prevalence of myopia in the Framingham Offspring Eye Study. Archives of ophthalmology 114: 326-32

Hofman A, van Duijn CM, Franco OH, Ikram MA, Janssen HL, Klaver CC, Kuipers EJ, Nijsten TE, Stricker BH, Tiemeier H, Uitterlinden AG, Vernooij MW, Witteman JC (2011) The Rotterdam Study: 2012 objectives and design update. Eur J Epidemiol 26: 657-86

Holle R, Happich M, Lowel H, Wichmann HE, Group MKS (2005) KORA--a research platform for population based health research. Gesundheitswesen 67 Suppl 1: S19-25

Hughes K, Aw TC, Kuperan P, Choo M (1997) Central obesity, insulin resistance, syndrome X, lipoprotein(a), and cardiovascular risk in Indians, Malays, and Chinese in Singapore. J Epidemiol Community Health 51: 394-9

Khor CC, Ramdas WD, Vithana EN, Cornes BK, Sim X, Tay WT, Saw SM, Zheng Y, Lavanya R, Wu R, Wang JJ, Mitchell P, Uitterlinden AG, Rivadeneira F, Teo YY, Chia KS, Seielstad M, Hibberd M, Vingerling JR, Klaver CC, Jansonius NM, Tai ES, Wong TY, van Duijn CM, Aung T (2011) Genome-wide association studies in Asians confirm the involvement of ATOH7 and TGFBR3,

and further identify CARD10 as a novel locus influencing optic disc area. Hum Mol Genet 20: 1864-72
Klein R, Lee KE, Gangnon RE, Klein BE (2010) The 25-year incidence of visual impairment in type 1 diabetes mellitus the wisconsin epidemiologic study of diabetic retinopathy. Ophthalmology 117: 63-70
Lavanya R, Jeganathan VS, Zheng Y, Raju P, Cheung N, Tai ES, Wang JJ, Lamoureux E, Mitchell P, Young TL, Cajucom-Uy H, Foster PJ, Aung T, Saw SM, Wong TY (2009) Methodology of the Singapore Indian Chinese Cohort (SICC) eye study: quantifying ethnic variations in the epidemiology of eye diseases in Asians. Ophthalmic Epidemiol 16: 325-36
Leibowitz HM, Krueger DE, Maunder LR, Milton RC, Kini MM, Kahn HA, Nickerson RJ, Pool J, Colton TL, Ganley JP, Loewenstein JI, Dawber TR (1980) The Framingham Eye Study monograph: An ophthalmological and epidemiological study of cataract, glaucoma, diabetic retinopathy, macular degeneration, and visual acuity in a general population of 2631 adults, 1973-1975. Surv Ophthalmol 24: 335-610
Li LJ, Cheung CY, Gazzard G, Chang L, Mitchell P, Wong TY, Saw SM (2011) Relationship of ocular biometry and retinal vascular caliber in preschoolers. Investigative ophthalmology & visual science 52: 9561-6
Li Y, Willer C, Sanna S, Abecasis G (2009) Genotype imputation. Annu Rev Genomics Hum Genet 10: 387-406
Louttit MD, Kopplin LJ, Igo RP, Jr., Fondran JR, Tagliaferri A, Bardenstein D, Aldave AJ, Croasdale CR, Price MO, Rosenwasser GO, Lass JH, Iyengar SK (2012) A multicenter study to map genes for Fuchs endothelial corneal dystrophy: baseline characteristics and heritability. Cornea 31: 26-35
Mackey DA, Mackinnon JR, Brown SA, Kearns LS, Ruddle JB, Sanfilippo PG, Sun C, Hammond CJ, Young TL, Martin NG, Hewitt AW (2009) Twins eye study in Tasmania (TEST): rationale and methodology to recruit and examine twins. Twin Res Hum Genet 12: 441-54
Oexle K, Ried JS, Hicks AA, Tanaka T, Hayward C, Bruegel M, Gogele M, Lichtner P, Muller-Myhsok B, Doring A, Illig T, Schwienbacher C, Minelli C, Pichler I, Fiedler GM, Thiery J, Rudan I, Wright AF, Campbell H, Ferrucci L, Bandinelli S, Pramstaller PP, Wichmann HE, Gieger C, Winkelmann J, Meitinger T (2011) Novel association to the proprotein convertase PCSK7 gene locus revealed by analysing soluble transferrin receptor (sTfR) levels. Hum Mol Genet 20: 1042-7
Pardo LM, MacKay I, Oostra B, van Duijn CM, Aulchenko YS (2005) The effect of genetic drift in a young genetically isolated population. Ann Hum Genet 69: 288-95
Parssinen O, Jauhonen HM, Kauppinen M, Kaprio J, Koskenvuo M, Rantanen T (2010) Heritability of spherical equivalent: a population-based twin study among 63- to 76-year-old female twins. Ophthalmology 117: 1908-11
Rahi JS, Cumberland PM, Peckham CS (2011) Myopia over the lifecourse: prevalence and early life influences in the 1958 British birth cohort. Ophthalmology 118: 797-804
Raitakari OT, Juonala M, Ronnemaa T, Keltikangas-Jarvinen L, Rasanen L, Pietikainen M, Hutri-Kahonen N, Taittonen L, Jokinen E, Marniemi J, Jula A, Telama R, Kahonen M, Lehtimaki T, Akerblom HK, Viikari JS (2008) Cohort profile: the cardiovascular risk in Young Finns Study. Int J Epidemiol 37: 1220-6
Saw SM, Chua WH, Hong CY, Wu HM, Chan WY, Chia KS, Stone RA, Tan D (2002a) Nearwork in early-onset myopia. Investigative ophthalmology & visual science 43: 332-9
Saw SM, Chua WH, Hong CY, Wu HM, Chia KS, Stone RA, Tan D (2002b) Height and its relationship to refraction and biometry parameters in Singapore Chinese children. Investigative ophthalmology & visual science 43: 1408-13
Saw SM, Tong L, Chua WH, Chia KS, Koh D, Tan DT, Katz J (2005) Incidence and progression of myopia in Singaporean school children. Investigative ophthalmology & visual science 46: 51-7
Sim X, Ong RT, Suo C, Tay WT, Liu J, Ng DP, Boehnke M, Chia KS, Wong TY, Seielstad M, Teo YY, Tai ES (2011) Transferability of type 2 diabetes implicated loci in multi-ethnic cohorts from Southeast Asia. PLoS Genet 7: e1001363

Smith EN, Chen W, Kahonen M, Kettunen J, Lehtimaki T, Peltonen L, Raitakari OT, Salem RM, Schork NJ, Shaw M, Srinivasan SR, Topol EJ, Viikari JS, Berenson GS, Murray SS (2010) Longitudinal genome-wide association of cardiovascular disease risk factors in the Bogalusa heart study. PLoS Genet 6

Spector TD, Williams FM (2006) The UK Adult Twin Registry (TwinsUK). Twin Res Hum Genet 9: 899-906

Steffens M, Lamina C, Illig T, Bettecken T, Vogler R, Entz P, Suk EK, Toliat MR, Klopp N, Caliebe A, Konig IR, Kohler K, Ludemann J, Diaz Lacava A, Fimmers R, Lichtner P, Ziegler A, Wolf A, Krawczak M, Nurnberg P, Hampe J, Schreiber S, Meitinger T, Wichmann HE, Roeder K, Wienker TF, Baur MP (2006) SNP-based analysis of genetic substructure in the German population. Hum Hered 62: 20-9

Vitart V, Bencic G, Hayward C, Herman JS, Huffman J, Campbell S, Bucan K, Zgaga L, Kolcic I, Polasek O, Campbell H, Wright A, Vatavuk Z, Rudan I (2010) Heritabilities of ocular biometrical traits in two croatian isolates with extended pedigrees. Invest Ophthalmol Vis Sci 51: 737-43

Vithana EN, Aung T, Khor CC, Cornes BK, Tay WT, Sim X, Lavanya R, Wu R, Zheng Y, Hibberd ML, Chia KS, Seielstad M, Goh LK, Saw SM, Tai ES, Wong TY (2011) Collagen-related genes influence the glaucoma risk factor, central corneal thickness. Hum Mol Genet 20: 649-58

Wichmann HE, Gieger C, Illig T, Group MKS (2005) KORA-gen--resource for population genetics, controls and a broad spectrum of disease phenotypes. Gesundheitswesen 67 Suppl 1: S26-30

Yazar S, Forward H, McKnight CM, Tan A, Soloshenko A, Oates SK, Ang W, Sherwin JC, Wood D, Mountain JA, Pennell CE, Hewitt AW, Mackey DA (2013) Raine eye health study: design, methodology and baseline prevalence of ophthalmic disease in a birth-cohort study of young adults. Ophthalmic genetics 34: 199-208

Zeller T, Wild P, Szymczak S, Rotival M, Schillert A, Castagne R, Maouche S, Germain M, Lackner K, Rossmann H, Eleftheriadis M, Sinning CR, Schnabel RB, Lubos E, Mennerich D, Rust W, Perret C, Proust C, Nicaud V, Loscalzo J, Hubner N, Tregouet D, Munzel T, Ziegler A, Tiret L, Blankenberg S, Cambien F (2010) Genetics and beyond--the transcriptome of human monocytes and disease susceptibility. PLoS One 5: e10693
